# Dynamic interaction network inference from longitudinal microbiome data

**DOI:** 10.1186/s40168-019-0660-3

**Published:** 2019-04-02

**Authors:** Jose Lugo-Martinez, Daniel Ruiz-Perez, Giri Narasimhan, Ziv Bar-Joseph

**Affiliations:** 1 Computational Biology Department, School of Computer Science, Carnegie Mellon University, 5000 Forbes Avenue, Pittsburgh, 15213 Pennsylvania USA; 2 Bioinformatics Research Group (BioRG), Florida International University, 11200 SW 8th Street, Miami, 33199 Florida USA; 3 Biomolecular Sciences Institute, Florida International University, Miami, 33199 Florida USA

**Keywords:** Dynamic interaction network inference, Longitudinal microbiome analysis, Microbial composition prediction, Dynamic Bayesian networks, Temporal alignment

## Abstract

**Background:**

Several studies have focused on the microbiota living in environmental niches including human body sites. In many of these studies, researchers collect longitudinal data with the goal of understanding not only just the composition of the microbiome but also the interactions between the different taxa. However, analysis of such data is challenging and very few methods have been developed to reconstruct dynamic models from time series microbiome data.

**Results:**

Here, we present a computational pipeline that enables the integration of data across individuals for the reconstruction of such models. Our pipeline starts by aligning the data collected for all individuals. The aligned profiles are then used to learn a dynamic Bayesian network which represents causal relationships between taxa and clinical variables. Testing our methods on three longitudinal microbiome data sets we show that our pipeline improve upon prior methods developed for this task. We also discuss the biological insights provided by the models which include several known and novel interactions. The extended CGBayesNets package is freely available under the MIT Open Source license agreement. The source code and documentation can be downloaded from https://github.com/jlugomar/longitudinal_microbiome_analysis_public.

**Conclusions:**

We propose a computational pipeline for analyzing longitudinal microbiome data. Our results provide evidence that microbiome alignments coupled with dynamic Bayesian networks improve predictive performance over previous methods and enhance our ability to infer biological relationships within the microbiome and between taxa and clinical factors.

**Electronic supplementary material:**

The online version of this article (10.1186/s40168-019-0660-3) contains supplementary material, which is available to authorized users.

## Background

Multiple efforts have attempted to study the microbiota living in environmental niches including human body sites. These microbial communities can play beneficial as well as harmful roles in their hosts and environments. For instance, microbes living in the human gut perform numerous vital functions for homeostasis ranging from harvesting essential nutrients to regulating and maintaining the immune system. Alternatively, a compositional imbalance known as dysbiosis can lead to a wide range of human diseases [1], and is linked to environmental problems such as harmful algal blooms [2].

While many studies profile several different types of microbial taxa, it is not easy in most cases to uncover the complex interactions within the microbiome and between taxa and clinical factors (e.g., gender, age, ethnicity). Microbiomes are inherently dynamic, thus, in order to fully reconstruct these interactions, we need to obtain and analyze longitudinal data [3]. Examples include characterizing temporal variation of the gut microbial communities from pre-term infants during the first weeks of life, and understanding responses of the vaginal microbiota to biological events such as menses. Even when such longitudinal data is collected, the ability to extract an accurate set of interactions from the data is still a major challenge.

To address this challenge, we need computational time-series tools that can handle data sets that may exhibit missing or noisy data and non-uniform sampling. Furthermore, a critical issue which naturally arises when dealing with longitudinal biological data is that of temporal rate variations. Given longitudinal samples from different individuals (for example, gut microbiome), we cannot expect that the rates in which interactions take place is exactly the same between these individuals. Issues including age, gender, external exposure, etc. may lead to faster or slower rates of change between individuals. Thus, to analyze longitudinal data across individuals, we need to first align the microbial data. Using the aligned profiles, we can next employ other methods to construct a model for the process being studied.

Most current approaches for analyzing longitudinal microbiome data focus on changes in outcomes over time [4, 5]. The main drawback of this approach is that individual microbiome entities are treated as independent outcomes, hence, potential relationships between these entities are ignored. An alternative approach involves the use of dynamical systems such as the generalized Lotka-Volterra (gLV) models [6–10]. While gLV and other dynamical systems can help in studying the stability of temporal bacterial communities, they are not well-suited for temporally sparse and non-uniform high-dimensional microbiome time series data (e.g., limited frequency and number of samples), as well as noisy data [3, 10]. Additionally, most of these methods eliminate any taxa whose relative abundance profile exhibits a zero entry (i.e., not present in a measurable amount at one or more of the measured time points. Finally, probabilistic graphical models (e.g., hidden Markov models, Kalman filters, and dynamic Bayesian networks) are machine learning tools which can effectively model dynamic processes, as well as discover causal interactions [11].

In this work, we first adapt statistical spline estimation and dynamic time-warping techniques for aligning time-series microbial data so that they can be integrated across individuals. We use the aligned data to learn a Dynamic Bayesian Network (DBN), where nodes represent microbial taxa, clinical conditions, or demographic factors and edges represent causal relationships between these entities. We evaluate our model by using multiple data sets comprised of the microbiota living in niches in the human body including the gastrointestinal tract, the urogenital tract, and the oral cavity. We show that models for these systems can accurately predict changes in taxa and that they greatly improve upon models constructed by prior methods. Finally, we characterize the biological relationships in the reconstructed microbial communities and discuss known and novel interactions discovered by these models.

## Methods

### Data sets

We collected multiple public longitudinal microbiome data sets for testing our method. Additional file 1: Table S1 summarizes each longitudinal microbiome data set used in this study, including the complete list of clinical features available.

**Infant gut microbiome** This data set was collected by La Rosa et al. [5]. They sequenced gut microbiomse from 58 pre-term infants in neonatal intensive care unit (NICU). The data was collected during the first 12 weeks of life (until discharged from NICU or deceased) sampled every day or two on average. Following analysis, 29 microbial taxa were reported across the 922 total infant gut microbiome measurements. In addition to the taxa information, this data set includes clinical and demographic information for example, gestational age at birth, post-conceptional age when sample was obtained, mode of delivery (C-section or vaginal), antibiotic use (percentage of days of life on antibiotic), and more (see Additional file 1: Table S1 for complete list of clinical features available).

**Vaginal microbiome** The vaginal microbiota data set was collected by Gajer et al. [4]. They studied 32 reproductive-age healthy women over a 16-week period. This longitudinal data set is comprised of 937 self-collected vaginal swabs and vaginal smears sampled two times a week. Analysis identified 330 bacterial taxa in the samples. The data also contains clinical and demographic attributes on the non-pregnant women such as Nugent score [12], menses duration, tampon usage, vaginal douching, sexual activity, race, and age. To test the alignment methods, we further sub-divided the microbial composition profiles of each subject by menstrual periods. This resulted in 119 time-series samples, an average of 3– 4 menstrual cycles per woman. Additional file 2: Figure S1a shows four sub-samples derived from an individual sample over the 16-week period along with corresponding menses information.

**Oral cavity microbiome** The oral cavity data was downloaded from the case-control study conducted by DiGiulio et al. [13] comprised of 40 pregnant women, 11 of whom delivered pre-term. Overall, they collected 3767 samples and identified a total of 1420 microbial taxa. Data was collected weekly during gestation and monthly after delivery from four body sites: vagina, distal gut, saliva, and tooth/gum. In addition to bacterial taxonomic composition, these data sets report clinical and demographic attributes which include gestational status, gestational or postpartum day when sample was collected, race, and ethnicity. In this paper, we solely focus on the tooth/gum samples during gestation from Caucasian women in the control group to reduce potential confounding factors. This restricted set contains 374 temporal samples from 18 pregnant women.

### Temporal alignment

As mentioned in the “Background” section, a challenge when comparing time series obtained from different individuals is the fact that while the overall process studied in these individuals may be similar, the *rates* of change may differ based on several factors (age, gender, other diseases, etc.). Thus, prior to modeling the relationships between the different taxa we first align the data sets between individuals by warping the time scale of each sample into the scale of another representative sample referred to as the *reference*. The goal of an alignment algorithm is to determine, for each individual *i*, a transformation function *τ*_*i*_(*t*) which takes as an input a reference time *t* and outputs the corresponding time for individual *i*. Using this function, we can compare corresponding values for all individuals sampled for the equivalent time point. This approach effectively sets the stage for accurate discovery of trends and patterns, hence, further disentangling the dynamic and temporal relationships between entities in the microbiome.

There are several possible options for selecting transformation function *τ*_*i*_. Most methods used to date rely on polynomial functions [14, 15]. Prior work on the analysis of gene expression data indicated that given the relatively small number of time points for each individual simpler functions tend to outperform more complicated ones [16]. Therefore, we used a first-degree polynomial: $\tau _{i}(t) = \frac {(t - b)}{a}$ as the alignment function for tackling the temporal alignment problem, where *a* and *b* are the parameters of the function.

### Data pre-processing

**Fig. 1:**
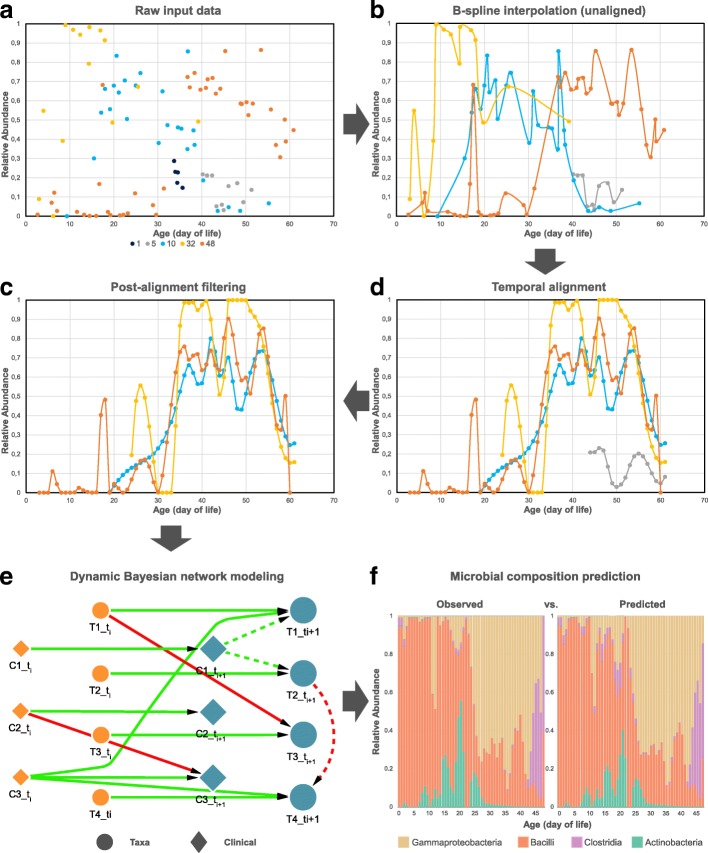
Schematic diagram illustrating the whole computational pipeline proposed in this work. Figure shows microbial taxa *Gammaproteobacteria* at each step in the pipeline from a set of five representative individual samples (subjects 1, 5, 10, 32, and 48) of the gut data set. **a** Input is raw relative abundance values for each sample measured at (potentially) non-uniform intervals even within the same subject. **b** Cubic B-spline curve for each individual sample. Sample corresponding to subject 1 (dark blue) contains less than pre-defined threshold for measured time points, thus, removed from further analysis. The remaining smoothed curves enable principled estimation of unobserved time points and interpolation at uniform intervals. **c** Temporal alignment of each individual sample against a selected reference sample (subject 48 shown in orange). **d** Post-alignment filtering of samples with alignment error higher than a pre-defined threshold. Sample corresponding to subject 5 (grey) discarded. **e** Learning a dynamic Bayesian network (DBN) structure and parameters. Let nodes (*T*_1_,*T*_2_,*T*_3_,*T*_4_) represent microbial taxa and (*C*_1_,*C*_2_,*C*_3_) represent clinical factors shown as circles and diamonds, respectively. Figure shows two consecutive time slices *t*_*i*_ and *t*_*i*+1_, where dotted lines connect nodes from the same time slice referred to as *intra edges*, and solid lines connect nodes between time slices referred to as *inter edges*. Biological relationships are inferred from edge parameters in the learned DBN which can be positive (green) or negative (red). **f** Original and predicted relative abundance across four gut taxa for subject 48 at sampling rate of 1 day. Performance is evaluated by average mean absolute error (MAE) between original and predicted abundance values (MAE =0.011)

Since alignment relies on continuous (polynomial) functions while the data is sampled at discrete intervals, the first step is to represent the sample data using continuous curves as shown by the transition from Fig. 1a to Fig. 1b. Following prior work [16], we use B-splines for fitting continuous curves to microbial composition time-series data, thus, enabling principled estimation of unobserved time points and interpolation at uniform intervals. To avoid overfitting, we removed any sample that had less than nine measured time points. The resulting pre-processed data is comprised of 48 individual samples of the infant gut, 116 sub-samples of the vaginal microbiota, and 15 pregnant women samples of the oral microbiome. We next estimated a cubic B-spline from the observed abundance profile for all taxa in remaining samples using *splrep* and *BSpline* from the Python function *scipy.interpolate*. In particular, *splrep* is used to find the B-spline representation (i.e., vector of knots, B-spline coefficients, and degree of the spline) of the observed abundance profile for each taxa, whereas *BSpline* is used to evaluate the value of the smoothing polynomial and its derivatives. Additional file 3: Figure S2 shows the original and cubic spline of a representative microbial taxa from a randomly selected individual sample across each data set.

### Aligning microbial taxon

To discuss the alignment algorithm, we first assume that a reference sample, to which all other samples would be aligned, is available. In the next section, we discuss how to choose such reference.

Formally, let $s_{r}^{j}(t)$ be the spline curve for microbial taxa *j* at time *t*∈[*t*_min_, *t*_max_] in the reference time-series sample *r*, where *t*_min_ and *t*_max_ denote the starting and ending time points of $s_{r}^{j}$, respectively. Similarly, let $s_{i}^{j}(t')$ be the spline for individual *i* in the set of samples to be warped for taxa *j* at time *t*^′^∈[*t*min′, *t*max′]. Next, analogously to Bar-Joseph et al. [14], the alignment error for microbial taxa *j* between $s_{r}^{j}$ and $s_{i}^{j}$ is defined as

$$e^{j} (r,i) = \frac{\int_{\alpha}^{\beta} \, \left(s_{i}^{j}(\tau_{i}(t)) - s_{r}^{j}(t)\right)^{2} dt}{\beta - \alpha}, $$

where $\alpha = \max \{t_{{\text {min}}}, \tau _{i}^{-1}(t'_{{\text {min}}})\}$ and $\beta = \min \left \{t_{{\text {max}}}, \tau _{i}^{-1}\left (t'_{{\text {max}}}\right)\right \}$ correspond to the starting and ending time points of the alignment interval. Observe that by smoothing the curves, it is possible to estimate the values at any intermediate time point in the alignment interval [*α*, *β*]. Finally, we define the microbiome alignment error for a microbial taxon of interest *S* between individual samples *r* and *i* as follows

$$E_{M} (r, i) = \sum\limits_{j \in S} e^{j} (r,i). $$

Given a reference *r* and microbial taxon *S*, the alignment algorithm task is to find parameters *a* and *b* that minimize *E*_*M*_ for each individual sample *i* in the data set subject to the constraints: *a*>0, *α*<*β* and $\frac {(\beta - \alpha)}{(t_{{\text {max}}} - t_{{\text {min}}})} \geq \epsilon $. The latter constraint enforces that the overlap between aligned interval [*α*, *β*] and reference interval [*t*_min_, *t*_max_] is at least *ε*; otherwise, trivial solutions (for example, no overlap leading to 0 error) would be selected. Here, we used *ε*=0.3 though results remain the same with larger values of *ε*. Figure 1c illustrates an aligned set of four samples where reference sample *r* is shown in orange. Alternatively, Additional file 2: Figure S1b shows the temporal alignment between the sub-samples of the vaginal microbiome sample shown in Figure S1a for the taxon *L. crispatus* using the first menstrual period sub-sample as reference (shown in orange).

### Selecting a reference sample

Finding an optimal reference that jointly minimizes the error for all samples (*E*_*M*_) is akin to solving a multiple alignment problem. Optimal solutions for such problems still require a runtime that is exponential in the number of samples [14] and so a heuristic approach was used instead. For this, we first find the best pairwise alignments via a grid-search parameter sweep between *a*∈(0,4] with increments of 0.01 and *b*∈[− 50,50] with increments of 0.5 in the linear alignment function *τ*_*i*_ previously described. It is important to note that this restricted search space for parameters *a* and *b* may lead to some sample pairs (*r*,*i*) without a temporal alignment because overlap constraint is not met. Additionally, we filtered out any microbial taxa *j*∈*S* for which the mean abundance in either $s_{r}^{j}$ or $s_{i}^{j}$ was less than 0.1*%*, or had zero variance over the originally sampled time points. Lastly, an optimal reference for each data set is determined by generating all possible pairwise alignments between samples. To select the best reference *r*^∗^, we employed the following criteria: (1) at least 90% of the individual samples are aligned to *r*^∗^, and (2) the alignment error *E*_*M*_ is minimized. We note that if no candidate reference meets these criteria, a commonly used heuristic for selecting *r*^∗^ picks the sample with the longest interval or highest number of measured time points.

**Abnormal or noisy samples filtering** As a post-processing step, we implemented a simple procedure which takes as input the resulting individual-wise alignments to identify and filter out abnormal and noisy samples. Given an aligned microbiome data set, we (1) computed the mean *μ* and standard deviation *δ* of the alignment error *E*_*M*_ across all aligned individual samples, and (2) removed all samples from an individual where *E*_*M*_>*μ*+(2×*δ*). Figure 1d shows the filtered set for the aligned taxa in the previous step (Fig. 1c). This analysis can both help to identify outliers and to improve the ability to accurately reconstruct models for interactions between taxa as shown in “Results” section.

**Taxon selection from alignment** As previously described, the microbiome alignment error *E*_*M*_ for a pairwise alignment is restricted to the set of microbial taxa *S* that contributed to the alignment. However, this set of microbes can vary for different pairwise alignments even with the same reference. Therefore, we focused on the subset of taxa that contributed to at least half of the pairwise alignments for the selected reference. Additional file 4: Table S2 lists alignment information for each data set such as reference sample, number of aligned samples, and selected taxa.

**Alignment simulation experiments** Since temporal alignment using splines does not guarantee convergence to a global minimum [14], we performed simulation studies to investigate the susceptibility to the non-uniqueness and local optima of the splines-based heuristic approach described at the beginning of this section. In particular, we first used the originally measured time points and observed abundance profile from three taxa of a representative individual sample in the gut data set as the reference sample. We then simulated 10 different individual samples as follows: for each individual sample, we manually warped the time points with randomly selected parameters *a* (scaling) and *b* (translation) such that *a*∈(0,4] and *b*∈[0,50]. We next added distinct percentage of Gaussian noise selected from {0,5,10,15,20,25} to the warped time points. To further test the robustness of splines, we also added Gaussian noise to the observed abundance profile of each taxa. Finally, we conducted three types of simulation experiments: (1) simulated noise-free warped time points for each individual sample but with noisy abundance profile, (2) simulated noise-free abundance profile but with noisy warped time points, and (3) noisy simulated warped time points with noisy abundance profiles.

From each simulation experiment, we aligned all simulated individual samples to the reference sample. We then computed and reported the mean absolute error (MAE) between the observed alignment parameters (i.e., *a* and *b*), as well as alignment error *E*_*M*_ on the aligned simulated data.

### Dynamic Bayesian network models

*Bayesian networks* (BNs) are a type of probabilistic graphical model consisting of a directed acyclic graph. In a BN model, the nodes correspond to random variables, and the directed edges correspond to potential conditional dependencies between them. The absence of an edge connecting two variables indicates independence or conditional independence between them. Conditional independence allows for a compact, factorized representation of the joint probability distribution [17].

*Dynamic Bayesian Networks* (DBNs) are BNs better suited for modeling relationships over temporal data. Instead of building different models across time steps, DBNs allow for a “generic slice” that shows transitions from a previous time point to the next time point, thus representing a generic temporal transition that can occur at any time during the computation. The incorporation of conditional dependence and independence is similar to that in BNs. DBNs have been widely used to model longitudinal data across many scientific domains, including speech [18, 19], biological [11, 20, 21], or economic sequences [22, 23].

More formally, a DBN is a directed acyclic graph where, at each *time slice* (or time instance), nodes correspond to random variables of interest (e.g., taxa, post-conceptional age, or Nugent score) and directed edges correspond to their conditional dependencies in the graph. These time slices are not modeled separately. Instead, a DBN contains edges connecting time slices known as *inter edges* that are repeated for each time point modeled as depicted in Fig. 1e. In summary, the model learns the transition probability from one time point to the next as a stationary conditional probability. DBNs are considered generative models, therefore, ideal for modeling the compositional interactions and dynamics of the microbiota given the first time point.

### Model construction

Using the aligned time series for the abundance of taxa, we next attempted to learn graphical models that provide information about the dependence of the abundance of taxa on the abundance of other taxa and clinical or demographic variables. Here, we use a “two-stage” DBN model in which only two slices are modeled and learned at a time. Throughout this paper, we will refer to the previous and current time points as *t*_*i*_ and *t*_*i*+1_, respectively. Fig. 1e illustrates a skeleton of the general structure of a two-stage DBN in the context of a longitudinal microbiome study. In this example, for each time slice, the nodes correspond to random variables of observed quantities for different microbial taxa (*T*_1_,*T*_2_,*T*_3_,*T*_4_) or clinical factors (*C*_1_,*C*_2_,*C*_3_) shown as circles and diamonds, respectively. These variables can be connected by intra edges (dotted lines) or inter edges (solid lines). In this DBN model, the abundance of a particular microbe in the current time slice is determined by parameters from both intra and inter edges, thus, modeling the complex interactions and dynamics between the entities in the microbial community.

Typically, analysis using DBNs is divided into two components: learning the network structure and parameters and inference on the network. The former can be further sub-divided into (i) structure learning which involves inferring from data the causal connections between nodes (i.e., learning the intra and inter edges) while avoiding overfitting the model, and (ii) parameter learning which involves learning the parameters of each intra and inter edge in a specific network structure. There are only a limited number of open software packages that support both learning and inference with DBNs [24, 25] in the presence of discrete and continuous variables. Here, we used the freely available CGBayesNets package [11, 24] for learning the network structure and performing inference for Conditional Gaussian Bayesian models [26]. While useful, CGBayesNets does not support several aspects of DBN learning including the use of intra edges, searching for a parent candidate set in the absence of prior information and more. We have thus extended the structure learning capabilities of CGBayesNets to include intra edges while learning network structures and implemented well-known network scoring functions for penalizing models based on the number of parameters such as Akaike Information Criterion (AIC) and Bayesian Information Criterion (BIC) [27].

**Learning DBN model parameters** Let *Θ* denote the set of parameters for the DBN and *G* denote a specific network structure over discrete and continuous variables in the microbiome study. In a similar manner to McGeachie et al. [11], we can decompose the joint distribution as

$$P(\Delta) F(\Psi|\Delta) = \prod_{x \in \Delta} p\left(x \, | \, \mathbf{Pa}^{G}(x)\right) \prod_{y \in \Psi} f\left(y \, | \, \mathbf{Pa}^{G}(y)\right) $$

where *P* denotes a set of conditional probability distributions over discrete variables *Δ*, *F* denotes a set of linear Gaussian conditional densities over continuous variables *Ψ*, and **Pa**^*G*^(*X*) denotes the set of parents for variable *X* in *G*. Since we are dealing with both continuous and discrete nodes in the DBN, in our method, continuous variables (i.e., microbial taxa compositions) are modeled using a Gaussian with the mean set based on a regression model over the set of continuous parents as follows

$$f(y \, | \, u_{1}, \cdots, u_{k}) \sim N\left(\lambda_{0} + \sum\limits_{i=1}^{k} \lambda_{i} \times u_{i}, \sigma^{2}\right) $$

where *u*_1_,⋯,*u*_*k*_ are continuous parents of *y*; *λ*_0_ is the intercept; *λ*_1_,⋯,*λ*_*k*_ are the corresponding regression coefficients for *u*_1_,⋯,*u*_*k*_; and *σ*^2^ is the standard deviation. We point out that if *y* has discrete parents then we need to compute coefficients $L = \{\lambda _{i}\}_{i=0}^{k}$ and standard deviation *σ*^2^ for each discrete parents configuration. For example, the conditional linear Gaussian density function for variable $T_{4\text {\_{\(t_{i+1}\)}}}$ in Fig. 1e denoted as $f\left (T_{4\text {\_{\(t_{i+1}\)}}} \, | \, T_{4\text {\_\(t_{i}\)}}, C_{3\text {\_\(t_{i}\)}}, T_{2\text {\_{\(t_{i+1}\)}}}\right)$ is modeled by

$$N\left(\lambda_{0} + \lambda_{1} \times T_{4\text{\_\(t_{i}\)}} + \lambda_{2} \times C_{3\text{\_\(t_{i}\)}} + \lambda_{3} \times T_{2\text{\_{\(t_{i+1}\)}}}, \sigma^{2}\right), $$

where *λ*_1_,*λ*_2_,*λ*_3_, and *σ*^2^ are the DBN model parameters. In general, given a longitudinal data set *D* and known structure *G*, we can directly infer the parameters *Θ* by maximizing the likelihood of the data given our regression model.

**Learning DBN structure** Learning the DBN structure can be expressed as finding the optimal structure and parameters

$$\max_{\Theta, G} P(D \, | \, \Theta, G) P(\Theta, G) = P(D, \Theta \, | \, G) P(G), $$

where *P*(*D* | *Θ*,*G*) is the likelihood of the data given the model. Intuitively, the likelihood increases as the number of valid parents **Pa**^*G*^(·) increases, thus, making it challenging to infer the most accurate model for data set *D*. Therefore, the goal is to effectively search over possible structures while using a function that penalizes overly complicated structures and protects from overfitting.

Here, we maximize *P*(*D*,*Θ* | *G*) for a given structure *G* using maximum likelihood estimation (MLE) coupled with BIC score instead of Bayesian Dirichlet equivalent sample-size uniform (BDeu) metric used in CGBayesNets. The BDeu score requires prior knowledge (i.e., equivalent sample size priors) which are typically arbitrarily set to 1; however, multiple studies have shown the sensitivity of BDeu to these parameters [28, 29], as well as the use of improper prior distributions [30]. Alternatively, BIC score does not depend on the prior over the parameters, thus, an ideal approach for scenarios where prior information is not available or difficult to obtain. Next, in order to maximize the full log-likelihood term we implemented a greedy hill-climbing algorithm. We initialize the structure by first connecting each taxa node at the previous time point (for example, $T_{1\text {\_\(t_{i}\)}}$ in Fig. 1e) to the corresponding taxa node at the next time point ($T_{1\text {\_{\(t_{i+1}\)}}}$ in Fig. 1e). We call this setting the *baseline* model since it ignores dependencies between taxa’s and only tries to infer taxa levels based on its levels in the previous time points. Next, we added nodes as parents of a specific node via intra or inter edges depending on which valid edge (i.e., no cycles) leads to the largest increase of the log-likelihood function beyond the global penalty incurred by adding the parameters as measured by the BIC^1^ score approximation

$$\text{BIC}(G, D) = \log P(D \, | \, \Theta, G) - \frac{d}{2} \log N, $$

where *d*=|*Θ*| is the number of DBN model parameters in *G*, and *N* is the number of time points in *D*. Additionally, we imposed an upper bound limit on the maximum number of possible parents (maxParents ∈{1,3,5}) for each bacterial node *X* (i.e., |**Pa**^*G*^(*X*)|≤maxParents).

### Inferring biological relationships

Microbial ecosystems are complex, often displaying a stunning diversity and a wide variety of relationships between community members. These biological relationships can be broadly divided into two categories: *beneficial* (including mutualism, commensalism, and obligate) or *harmful* (including competition, amensalism, and parasitism). Although the longitudinal data sets considered in this study do not provide enough information to further sub-categorize each biological relationship (e.g., mutualism vs. commensalism), we use the learned DBN model from each microbiome data set and inspect each interaction as a means for inferring simple to increasingly complex relationships. For example, consider variable $T_{4\text {\_\(t_{i}\)}}$ in Fig. 1e. Given that *t*_*i*_ and *t*_*i*+1_ represent the previous time point and the current time point (respectively), the possible inference in this case is as follows: edges from $T_{4\text {\_\(t_{i}\)}}$ and $C_{3\text {\_\(t_{i}\)}}$ (inter edges) and from $T_{2\text {\_{\(t_{i+1}\)}}}$ (intra edge) suggest the existence of a temporal relationship in which the abundance of taxa *T*_4_ at a previous time instant and abundance of taxa *T*_2_ at the current time instant, as well as condition *C*_3_ from the previous time instant impact the abundance of *T*_4_ at the current time. We previously stated that $f(T_{4\text {\_{\(t_{i+1}\)}}} \, | \, T_{4\text {\_\(t_{i}\)}}, C_{3\text {\_\(t_{i}\)}}, T_{2\text {\_{\(t_{i+1}\)}}})$ is modeled by $\phantom {\dot {i}\!}N(\lambda _{0} + \lambda _{1} \times T_{4\text {\_\(t_{i}\)}} + \lambda _{2} \times C_{3\text {\_\(t_{i}\)}} + \lambda _{3} \times T_{2\text {\_{\(t_{i+1}\)}}}, \sigma ^{2})$. Therefore, inspecting the regression coefficients *λ*_1_,*λ*_2_,*λ*_3_ immediately suggests whether the impact is positive or negative. In this example, the regression coefficients *λ*_1_,*λ*_2_ are positive (*λ*_1_,*λ*_2_>0) while coefficient *λ*_3_ is negative (*λ*_3_<0), thus, variables $T_{4\text {\_\(t_{i}\)}}$ and $C_{3\text {\_\(t_{i}\)}}$ exhibit positive relationships with microbial taxa $T_{4\text {\_{\(t_{i+1}\)}}}$ shown as green edges in Fig. 1e, whereas taxa $T_{2\text {\_\(t_{i}\)}}$ exhibits a negative interaction with $T_{4\text {\_{\(t_{i+1}\)}}}$ shown as a red edge (Fig. 1e). This simple analytic approach enables us to annotate each biological relationship with directional information.

### Network visualization

All the bootstrap networks^2^ shown are visualized using *Cytoscape* [31] version 3.6.0, using Attribute Circle Layout with Organic Edge Router. An in-house script is used to generate a custom style XML file for each network, encoding multiple properties of the underlying graph. Among these properties, the regression coefficients corresponding to edge thickness were normalized as follows: let *y* be a microbial taxa node with continuous taxa parents *u*_1_,⋯,*u*_*k*_ modeled by

$$f(y \, | \, u_{1}, \cdots, u_{k}) \sim N\left(\lambda_{0} + \sum\limits_{i=1}^{k} \lambda_{i} \times u_{i}, \sigma^{2}\right) $$

where *λ*_1_,⋯,*λ*_*k*_ are the corresponding regression coefficients for *u*_1_,⋯,*u*_*k*_ as previously described in this section. The normalized regression coefficients $\left \{\lambda ^{N}_{i}\right \}_{i=1}^{k}$ are defined as

$${\lambda^{N}_{i}} = \frac{\lambda_{i} \times \bar{u_{i}}}{{\sum\nolimits}_{j=1}^{k} \left|{\lambda_{j} \times \bar{u_{j}}}\right|}, $$

where $\bar {u_{i}}$ is the mean abundance of taxa *u*_*i*_ across all samples.

## Results

Figure 1 presents a schematic diagram illustrating the whole computational pipeline we developed for aligning and learning DBNs for microbiome and clinical data. We start by estimating a cubic spline from the observed abundance profile of each taxa (Fig. 1b). Next, we determine an alignment which allows us to directly compare temporal data across individuals (Fig. 1c), as well as filter out abnormal and noisy samples (Fig. 1d). Finally, we use the aligned data to learn causal dynamic models that provide information about interactions between taxa, their impact, and the impact of clinical variables on taxa levels over time (Fig. 1e–f).

We applied our methods to study longitudinal data sets from three human microbiome niches: infant gut, vagina, and oral cavity (see “Methods” section for full descriptions). In addition to the differences in the taxa they profile, these data sets vary in the number of subjects profiled (ranging from 15 to 48), in the number of time points they collected, the overall number of samples and time series that were studied, etc. Thus, they provide a good set to test the generality of our methods and their usefulness in different microbiome studies.

### Temporal alignments

Below, we discuss in detail the improved accuracy of the learned dynamic models due to use of *temporal alignments*. However, even before using them for our models, we wanted to verify our splines-based heuristic alignment approach, as well as test whether the alignment results agree with biological knowledge.

**Simulation experiments** To investigate whether our splines-based greedy alignment approach is able to identify good solutions, we performed several simulation experiments (described in “Methods” section). In summary, we simulated data for 10 individual samples and aligned them against a reference sample. We next computed the alignment accuracy (MAE) between the observed and expected alignment parameters (i.e., *a* and *b*), and alignment error *E*_*M*_ on the simulated data. These results are shown in Additional file 5: Figure S3, where the average error for alignment parameter *a* ranges between 0.030− 0.035 at 5% noise up to 0.24− 0.35 at 25% noise across all simulation experiments. Alternatively, the average error for alignment parameter *b* ranges between 0.25− 0.30 at 5% noise up to 4.5− 6.2 at 25% noise across all three experiments. Finally, the alignment error *E*_*M*_ is at most 7% at 25% noise which indicates large agreement between the aligned samples. Overall, these simulation results provide evidence that the proposed greedy search method is able to find good alignments, thus, supporting our prior assumptions as well as the use of B-splines.

**Infant gut alignments capture gestational age at birth** To test whether the alignment results agree with biological knowledge, we used the infant gut data. Infant gut microbiota goes through a patterned shift in dominance between three bacterial populations (*Bacilli* to *Gammaproteobacteria* to *Clostridia*) in the weeks immediately following birth. La Rosa et al. [5] reported that the rate of change is dependent on maturation of the infant highlighting the importance of post-conceptional age as opposed to day of life when analyzing bacterial composition dynamics in pre-term infants. We found that our alignment method is able to capture this rate of change without explicitly using gestational or post-conceptional age.

**Fig. 2:**
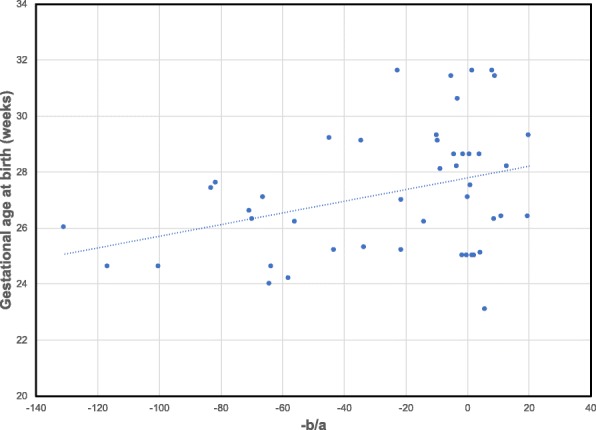
Relationship between alignment parameters and gestational age at birth. Figure shows the relationship between alignment parameters *a* and *b* and gestational age at birth (measured in weeks) for the aligned infant gut microbiome data set. Each blue dot represent an aligned infant sample *i* where *x*-axis shows $\frac {-b}{a}$ from transformation function $\tau _{i} (t) = \frac {(t - b)}{a}$ and *y*-axis shows the gestational age at birth of infant *i*. Pearson correlation coefficient = 0.35

Figure 2 shows the relationship between alignment parameters *a* and *b* (from the transformation function $\tau _{i}(t) = \frac {(t - b)}{a}$ described in “Methods” section) and the gestational age at birth for each infant in the gut microbiome data set. Each aligned infant sample is represented by a blue circle where the *x*-axis shows $\frac {-b}{a}$ and *y*-axis shows the gestational age at birth. As can be seen, the alignment parameters are reasonably well correlated with gestational age at birth (Pearson’s correlation coefficient = 0.35) indicating that this method can indeed be used to infer differences in rates between individuals.

### Resulting dynamic Bayesian network models

**Fig. 3:**
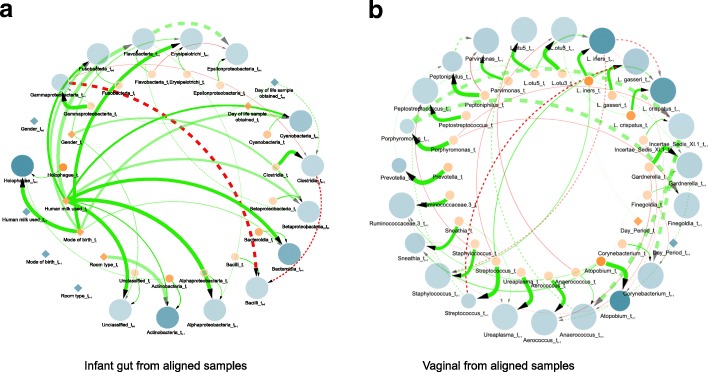
Learned dynamic Bayesian network for infant gut and vaginal microbiomes derived from aligned samples. Figure shows two consecutive time slices *t*_*i*_ (orange) and *t*_*i*+1_ (blue), where nodes are either microbial taxa (circles) or clinical/demographic factors (diamonds). Nodes size is proportional to in-degree whereas taxa nodes transparency indicates mean abundance. Additionally, dotted lines denote *intra edges* (i.e., directed links between nodes in same time slice) whereas solid lines denote *inter edges* (i.e., directed links between nodes in different time slices). Edge color indicates positive (green) or negative (red) temporal influence and edge transparency indicates strength of bootstrap support. Edge thickness indicates statistical influence of regression coefficient as described in network visualization. **a** Learned DBN for the aligned infant gut microbiome data at a sampling rate of 3 days and maxParents = 3. **b** Learned DBN for the aligned vaginal microbiome data at a sampling rate of 3 days and maxParents = 3

We next applied the full pipeline to learn DBNs from the three microbiome data sets under study. In particular, we use longitudinal data sets from three human microbiome niches: infant gut, vaginal, and oral cavity as described in “Methods” section. In this section, we highlight the overall characteristics of the learned DBN for each aligned and filtered microbiome data set (Fig. 3 and Additional file 6: Figure S4a). By contrast, we also show the learned DBN for each unaligned and filtered microbiome data set in Additional file 6: Figure S4b and Additional file 7: Figure S5. In all these figures, the nodes represent taxa and clinical (or demographic) variables and the directed edges represent temporal relationships between them. Several triangles were also observed in the networks. In some of the triangles, directed edges to a given node were linked from both time slices of another variable. We will refer to these as *directed triangles*.

**Infant gut** The learned DBN model for the infant gut microbiota data set at a sampling rate of 3 days and maxParents = 3 was computed. It contains 19 nodes per time slice (14 microbial taxa, 4 clinical, and 1 demographic variable nodes) and 39 directed edges (31 inter edges and 8 intra edges) with no directed triangles as shown in Fig. 3a. Since we only learn temporal conditional dependence (i.e., incoming edges) for taxa nodes at time slice *i*+1, the maximum number of possible edges is 14×maxParents = 42; thus, most of the taxa nodes (11 out of 14) have reached the maximum number of parents allowed (i.e., *maxParents* = 3). Additionally, the majority of these temporal relationships are between microbial taxa. In particular, the model includes several interactions between the key colonizers of the premature infant gut: *Bacilli*, *Clostridia*, and *Gammaproteobacteria*. Furthermore, the only negative interactions learned by the model comprise these microbes which are directly involved in the progression of the infant gut microbiota. Also, the nodes for gestational age at birth and post-conceptional age at birth are not shown because they are isolated from the rest of the network, without any single edge. Overall, these trends strongly suggest that the DBN is capturing biologically relevant interactions between taxa.

**Vaginal** As with the gut microbiome data set, we learned a DBN model for the vaginal microbiome data at a sampling rate of 3 days and maxParents = 3 (Fig. 3b). The resulting DBN is comprised of 24 nodes per time instance (23 taxa and 1 clinical) and 58 edges (40 inter edges and 18 intra edges). Additionally, 12 directed triangles involving taxa nodes were observed. In preliminary analyses, additional clinical and demographic attributes (e.g., Nugent category, race, and age group) resulted in networks with these variables connected to all taxa nodes, thus, removed from further analysis. Specifically, we estimated the degree of overfitting of these variables by learning and testing DBN models with and without them. This resulted in the DBN shown in Fig. 3b which exhibited lowest generalization error. In this case, the maximum number of potential edges between bacterial nodes is 24×maxParents = 72; however, only 16 out of 24 taxa nodes reached the threshold on the maximum number of parents. Among all the 58 edges, only 1 interaction *Day_Period_* *t*_*i*+1_ to *L. iners_* *t*_*i*+1_ involves a clinical node whereas the remaining 57 edges (including 15 negative interactions) captured temporal relationships among microbial taxa. This mixture of positive and negative interactions between taxa provides evidence of the DBNs ability to capture the complex relationships and temporal dynamics of the vaginal microbiota.

**Oral cavity** We learned a DBN with the longitudinal tooth/gum microbiome data set with a sampling rate of 7 days and maxParents = 3. Additional file 6: Figure S4a shows the learned DBN which contains 20 nodes for each time slice (19 taxa and 1 clinical) and 52 edges (33 inter edges and 19 intra edges) out of 57 possible edges. In addition, 2 directed triangles were observed involving taxa nodes. Here, the DBN model includes multiple positive and negative interactions among early colonizers (e.g., *Veillonella* and *H. parainfluenzae*) and late colonizers (e.g., *Porphyromonas*) of the oral microbiota which are supported by previous experimental studies [32].

### Comparisons to prior methods

**Fig. 4:**
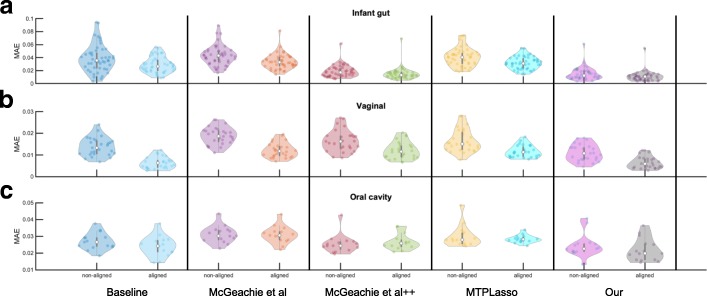
Comparison of average predictive accuracy between methods on the filtered data sets. Figure shows violin plots of the MAE distributions of our proposed DBN models against a baseline method and previously published approaches for a sampling rate that most closely resembles the originally measured time points. Additionally, each method is run on the non-aligned and aligned data sets. **a** Performance results for infant gut microbiome data for sampling rate of 3 days. **b** Performance results for vaginal microbiome data for sampling rate of 3 days. **c** Performance results for oral cavity microbiome data for sampling rate of 7 days

To evaluate the accuracy of our pipeline and to compare them to models reconstructed by prior methods published in the literature [11, 33], we used a per-subject cross-validation with the goal of predicting microbial taxon abundances using the learned models. In each iteration, the longitudinal microbial abundance profile of a single subject was selected as the test set, and the remaining profiles were used for building the network and learning model parameters. Next, starting from the second time point, we used the learned model to predict an abundance value for every taxa in the test set at each time point using the previous and current time points. Predicted values were normalized to represent relative abundance of each taxa across the microbial community of interest. Finally, we measured the average predictive accuracy by computing the MAE for the selected taxon in the network. We repeated this process (learning the models and predicting based on them) for several different sampling rates, which ranged from 1 up to 28 days depending on the data set. The original and predicted microbial abundance profiles can be compared as shown in Fig. 1f. The average MAE for predictions on the three data sets are summarized in Additional file 8: Table S3. Furthermore, Fig. 4 and Additional file 9: Figure S6 show violin and bar plots of the MAE distributions for ten different methods on each data set, respectively. Along with two of our DBNs (one with and one without alignments), four methods with and four without alignments were compared. These are further described below.

First, we compared the DBN strategy to a naive (baseline) approach. This baseline approach makes the trivial prediction that the abundance value for each taxa *A* at any given point is exactly equal to the abundance measured at the previous time point. Given that measured abundances are continuous variables, this turns out to be an extremely competitive method and performs better than most prior methods for the data sets we tested on. Next, we compared our DBNs to three other methods suggested for modeling interactions among taxa: (a) McGeachie et al. [11] developed a different DBN model where network learning is estimated from the BDeu scoring metric [24] (instead of MLE), (b) McGeachie et al.++ an in-house implementation that extends McGeachie et al.’s method to allow for intra edges during structure learning, and (c) MTPLasso [33] that models time-series microbial data using a gLV model. In all cases, we used the default parameters as provided in the original publications.

As can be seen by Table S3 and Figure S6, our method outperforms the baseline and previous methods for the infant gut data. It also performs favorably when compared to baseline on the other two data sets. Temporal alignments improved the predictive performance over unaligned samples across gut and vaginal microbiomes by about 1–4 percentage points. In particular, a two-tailed *t* test indicates significant (denoted by *) performance improvements for most sampling rates (infant gut: *p* value = 0.043* for 1 day, *p* value = 0.034* for 3 days, *p* value = 0.109 for 5 days, and *p* value < 1.00E-05* for 7 days; vaginal: *p* value < 1.00E-06* for 1 day, *p* value < 1.00E-05* for 3 days, *p* value = 5.50E-05* for 5 days, *p* value = 3.10E-03* for 7 days, and *p* value= 0.097 for 14 days). On the other hand, alignments did not show significant predictive performance improvements on the oral data set and is consistent with previous analysis on the same data set [13]. Surprisingly, the simple baseline approach outperforms all previously published methods: McGeachie et al. [11] and MTPLasso [33] across the three data sets. Finally, Fig. 4 shows violin plots of the MAE results for each data set across a sampling rate that most closely resembles the originally measured time points.

### Anomaly detection using alignment

When analyzing large cohorts of microbiome data, it is important to implement a strategy to remove outliers as these can affect our ability to generalize from the collected data. As discussed in “Methods” section, we can use our alignment error *E*_*M*_ score to identify such subjects and remove them prior to modeling. In the context of the gut data set, this resulted in the identification of two infant samples: subjects 5 and 55 (highlighted in red within Additional file 10: Figure S7a) which are likely processing errors, contaminated samples, or just natural anomalies. Sample 55 has been previously identified as a likely abruption event by McGeachie et al. [11] using a different approach. Similarly, Additional file 10: Figure S7b shows the distribution of alignment errors *E*_*M*_ for the vaginal microbiome data. In this case, we remove 6 sub-samples from 4 different women (highlighted in red). We note that there were no outliers identified in the oral cavity microbiome data set. When learning DBNs following the filtering we obtain even better models. Additional file 11: Figure S8 compares the average MAE results of our proposed DBN model between the unfiltered and filtered samples for the gut and vaginal data sets. As can be seen, a large performance improvement is observed for the gut data while a slight improvement is observed for the vaginal data when removing the outliers. These results suggest that even though the method uses less data to learn the models, the models that it does learn are more accurate.

## Discussion

### The power of temporal alignments

We developed a pipeline for the analysis of longitudinal microbiome data and applied it to three data sets profiling different human body parts. To evaluate the reconstructed networks we used them to predict changes in taxa abundance over time. Interestingly, ours is the first method to improve upon a naive baseline (Additional file 9: Figure S6). While this does not fully validate the accuracy of the models, it does mean that the additional interactions determined by our method contribute to the ability to infer future changes and so at least some are likely true.

As part of our pipeline, we perform temporal alignment. While ground truth for alignments is usually hard to determine, in one of the data sets we analyzed we could compare the alignment results to external information to test its usefulness. In the context of the infant gut data, it has been shown that using day of life as the independent variable hinders the identification of associations between bacterial composition and day of sampling. Therefore, previous work have re-analyzed the premature gut microbiota with post-conceptional age, uncovering biologically relevant relationships [5]. By using alignment we were able to correct for this difference without the need to rely on the external age information. In addition to the results presented in Fig. 2, the learned DBN in Fig. 3a does not show any relationships to post-conceptional age or gestational age at birth indicating that our alignment was able to successfully compensate for. By contrast, the learned DBN from unaligned samples in Additional file 7: Figure S5a shows relationships to post-conceptional age. While for this data such correction could have been made using post-conceptional age, in other cases the reason for the rate change may not be obvious and without alignment it would be hard to account for such hidden effects.

### Uncovering biological relationships

We next discuss in more detail the learned DBN models.

**Infant gut** As mentioned in “Results” section, the only negative relationships identified supports the known colonization order, that is, a shift in dominance from *Bacilli* to *Gammaproteobacteria* to *Clostridia*) [5], as the infant goes through the first several weeks of life. These edges show incoming negative relationships to *Bacilli* from *Gammaproteobacteria* and *Clostridia*. In particular, an increase in the abundance of the parents is associated with a decrease in the abundance of the child. The negative edge from *Gammaproteobacteria* to *Clostridia* agrees with previous findings where *Clostridia*’s abundance is found to increase at a gradual rate until it peaks at post-conceptional age between 33 and 36 weeks whereas *Gammaproteobacteria* decreases as infants age [5, 11]. It is important to note that this negative edge from *Gammaproteobacteria* to *Clostridia* is not found in the learned DBN from unaligned samples (Additional file 7: Figure S5a). This relationship is also confirmed by the edges from *Day of life* to *Gammaproteobacteria* and *Clostridia* (Fig. 3b). Moreover, the DBN model indicates a relationship between breastfeeding and *Actinobacteria*, *Bacteroidia*, and *Alphaproteobacteria*. These bacteria are known to be present in breast milk which is known to heavily influence and shape the infant gut microbiome [34].

**Vaginal** It has been established that microbial composition can change dramatically during the menses cycle and later return to a ‘stable’ state before the next menstrual period [35, 36]. Previous studies have identified a subset of individuals in this data set as exhibiting a microbial composition dominated by *L. crispatus* with a notable increase of *L. iners* around the start of each menstrual period [4, 35] (Additional file 2: Figure S1a). These interactions were also captured by the learned DBN model in the form of a directed triangle involving *L. crispatus* and *L. iners* (Fig. 3b). The edge from the *Day Period* to *L. iners* strengthens this relationship, which is not present in the learned DBN from unaligned vaginal sub-samples (Additional file 7: Figure S5b). On the other hand, subjects from another group were characterized as dominated by *L. gasseri* coupled with shifts to *Streptococcus* during menstruation [4]. These relationships were also captured by the DBN. Furthermore, while *L. iners* has a lower protective value than the other *Lactobacillus* [37], the negative edge between *L. iners* and *Atopobium* suggests a relationship related to environment protection. Also, the positive edge from *Atopobium* to *Gardnerella* is supported by the synergy observed between these two taxa in bacterial vaginosis [38]. Although many of these microbial relationships are also observed in the learned DBN from unaligned sub-samples, there are some biological relationships which cannot be found within the DBN derived without alignments. However, given our limited understanding of the interactions within the vaginal microbiome, we cannot determine whether or not these previously unseen interactions are biologically relevant. Finally, it is worth highlighting that the shifts and composition of the vaginal microbiome vary considerably between each women [4, 36].

**Oral** For oral microbiomes, several *Streptococcus* species, including *S. oralis*, *S. mitis*, *S. gordonii*, and *S. sanguis* are well known as early colonizers lying close to the tooth pellicle [32]. While our learned DBNs (Additional file 6: Figure S4) cannot identify specific species, it suggests interactions between some species of *Streptococcus* and other later colonizers in the oral microbiome such as *Porphyromonas* and *Prevotella*. The learned DBN derived from aligned tooth/gum samples also provided novel predictions, for example, taxa *Granulicatella* is interacting with *Veilonella*. Furthermore, there are other microbial relationships uniquely observed on each DBN which are also potentially interesting.

### Triangles in DBNs

An interesting aspect shared by all of the DBNs discussed above is the fact that they contain triangles or feed-forward loops. In particular, many of these directed triangles are created from nodes representing both time slices of another variable, but with different signs (one positive and the other negative). For example, microbial taxa *L. crispatus* displays a directed triangle with another taxa *L. iners* in the vaginal DBN (Fig. 3b). In this triangle, positive edges from *L. iners_* *t*_*i*_ interact with *L. iners_* *t*_*i*+1_ and *L. crispatus_* *t*_*i*+1_ whereas a negative edge connects *L. iners_* *t*_*i*+1_ to *L. crispatus_* *t*_*i*+1_.

The triangles in the DBNs represent a relationship where the abundance of a child node cannot be solely determined from the abundance of a parent at one time slice. Instead, information from both the previous and the current time slices is needed. This can be interpreted as implying that the child node is associated with the *change* of the abundance values of the parents rather than with the absolute values which each node represents.

### Limitation and future work

While our pipeline of alignment followed by DBN learning successfully reconstructed models for the data sets we looked at, it is important to understand the limitation of the approach. First, given the complexity of aligning a large number of individuals, our alignment method is based on a greedy algorithm, thus, it is not guaranteed to obtain the optimal result. Even if the alignment procedure is successful, the DBN may not be able to reflect the correct interactions between taxa. Issues related to sampling rates can impact the accuracy of the DBN (missing important intermediate interactions) while on the other hand if not enough data is available the model can overfit and predict non-existent interactions.

Given these limitations, we would attempt to improve the alignment method and its guarantees in future work. We are also interested in studying the ability of our procedure to integrate additional molecular longitudinal information including gene expression and metabolomics data which some studies are now collecting in addition to the taxa abundance data [39]. We believe that our approach for integrating information across individual in order to learn dynamic models would be useful for several ongoing and future studies.

## Conclusions

In this paper, we propose a novel approach to the analysis of longitudinal microbiome data sets using dynamic Bayesian networks with the goal of eliciting temporal relationships between various taxonomic entities and other clinical factors describing the microbiome. The novelty of our approach lies in the use of temporal alignments to normalize the differences in pace of biological processes inherent within different subjects. Additionally, the alignment algorithm can be used to filter out abruption events or noisy samples. Our results show that microbiome alignments improve predictive performance over previous methods and enhance our ability to infer known and potentially novel biological and environmental relationships between the various entities of a microbiome and the other clinical and demographic factors that describe the microbiome.

## Additional files

Additional file 1 **Table S1.** Summary of longitudinal microbiome data sets. For each data set, we show the total number of individuals *n*_*i*_, number of time series samples *n*_*s*_, number of microbial taxa reported *n*_*t*_, original sampling rate, and list of clinical attributes available. (PDF 57 kb)

Additional file 2 **Figure S1.** Representative vaginal microbiome sample for subject 28 over the 16-week period. **a** Relative abundance profile of six vaginal taxa for subject 48 over 16 weeks annotated with menses information. The vertical black lines correspond to the division of sub-samples based on menstrual periods (i.e., 4 sub-samples). Note the interpolated shift in dominance during menses between *L. crispatus* and *L. iners*. **b** | Temporal alignment between the sub-samples from subject 28 time-series data for taxa *L. crispatus* using the first menstrual period sub-sample as reference (shown in orange). Figure also shows abundance profile of *L. crispatus* for each sub-sample before (left) and after (right) alignment. (PDF 431 kb)

Additional file 3 **Figure S2.** Original and cubic spline of the abundance profile of a representative microbial taxa for each data set. Figure shows the original abundance values vs. the cubic B-spline curve for a representative taxa profile from a randomly selected individual sample across each data set. **a***Bacilli* from the infant gut microbiome. **b***L. iners* from the vaginal microbiome. **c***Prevotella* from the oral cavity microbiome. (PDF 39 kb)

Additional file 4 **Table S2.** Summary of alignment information. For each data set, we show reference sample, number of aligned samples *n*_*r*_, and selected taxa. (PDF 61 kb)

Additional file 5 **Figure S3.** Temporal alignment accuracy on simulated data. Figure shows MAE alongside standard deviation for alignment parameters *a* and *b*, as well as alignment error *E*_*M*_ using our heuristic alignment approach as a function of percentage of Gaussian noise. **a** Alignment performance on simulation experiment 1. **b** Alignment performance on simulation experiment 2. **c** Alignment performance on simulation experiment 3. (PDF 33 kb)

Additional file 6 **Figure S4.** Learned dynamic Bayesian network of the oral microbiome derived from unaligned and aligned tooth/gum samples. Figure shows two consecutive time slices *t*_*i*_ (orange) and *t*_*i*+1_ (blue), where nodes are either microbial taxa (circles) or clinical factors (diamonds). Nodes size is proportional to in-degree whereas taxa nodes transparency indicates mean abundance. Additionally, dotted lines denote *intra edges* whereas solid lines denote *inter edges*. Edges color indicates positive (green) or negative (red) temporal influence, and edge transparency indicates strength of bootstrap value. Edge thickness indicates statistical influence of regression coefficient as described in network visualization. **a** Learned DBN for the aligned oral microbiome data at a sampling rate of 7 days and maxParents = 3. **b** Learned DBN for the unaligned oral microbiome data at a sampling rate of 7 days and maxParents = 3. (PDF 55 kb)

Additional file 7 **Figure S5.** Learned dynamic Bayesian network for gut and vaginal microbiomes derived from unaligned samples. Figure shows two consecutive time slices *t*_*i*_ (orange) and *t*_*i*+1_ (blue), where nodes are either microbial taxa (circles) or clinical/demographic factors (diamonds). Nodes size is proportional to in-degree whereas taxa nodes transparency indicates mean abundance. Additionally, dotted lines denote *intra edges* (i.e., directed links between nodes in same time slice) whereas solid lines denote *inter edges* (i.e., directed links between nodes in different time slices). Edge color indicates positive (green) or negative (red) temporal influence, and edge transparency indicates strength of bootstrap support. Edge thickness indicates statistical influence of regression coefficient as described in network visualization. **a** Learned DBN for the unaligned infant gut microbiome data at a sampling rate of 3 days and maxParents = 3. **b** Learned DBN for the unaligned vaginal microbiome data at a sampling rate of 3 days and maxParents = 3. (PDF 56 kb)

Additional file 8 **Table S3.** Summary of average predictive accuracy and standard deviation between methods on the filtered data sets. For each data set, we list the average MAE and standard deviation (presented as percentage) of our proposed DBN models against a baseline method and previously published approaches across different sampling rates. Additionally, each method is run on the non-aligned and aligned data sets. The highest predictive accuracy for each sampling rate is shown in boldface. (PDF 60 kb)

Additional file 9 **Figure S6.** Comparison of average predictive accuracy and standard deviation between methods on the filtered data sets. Figure shows the average MAE and standard deviation of our proposed DBN models against a baseline method and previously published approaches as a function of sampling rates. Additionally, each method is run on the unaligned and aligned data sets. **a** Performance results for infant gut microbiome data. **b** Performance results for vaginal microbiome data. **c** Performance results for oral cavity microbiome data. (PDF 51 kb)

Additional file 10 **Figure S7.** Distribution of microbiome alignment error *E*_*M*_ for infant gut and vaginal data sets. **a** *E*_*M*_ scores for 47 infant gut samples aligned against a common reference gut sample. **b** *E*_*M*_ scores for 112 vaginal microbiome sub-samples aligned against an optimal reference sub-sample. In both panels, the scores highlighted in red represent samples with *E*_*M*_ at least two standard deviations away from the mean of the distribution of microbiome alignment errors, thus, identified as outliers and removed. (PDF 21 kb)

Additional file 11 **Figure S8.** Effect of outliers on average predictive accuracy from aligned data sets. Figure shows the average MAE for our proposed DBN model and baseline method as a function of sampling rates before (labeled as *unfiltered*) and after (labeled as *filtered*) removal of outliers. **a** Performance results for infant gut microbiome data. **b** Performance results for vaginal microbiome data. (PDF 30 kb)

## Abbreviations

AIC: Akaike information criterion
BDeu: Bayesian Dirichlet equivalent sample-size uniform
BIC: Bayesian information criterion
DBN: Dynamic Bayesian network
gLV: Generalized Lotka-Volterra
MLE: Maximum likelihood estimation
MAE: Mean absolute error
NICU: Neonatal intensive care unit

## Footnotes

1 We also computed AIC score (i.e., AIC(*G*,*D*)= log*P*(*D* | *Θ*,*G*)−*d*) but it was consistently outperformed by BIC score.

2 For each data set, we ran 500 bootstrap realizations and only reported edges with bootstrap support of at least 50% in the consensus DBN.
