# Supplementary material for: Dynamic interaction network inference from longitudinal microbiome data
Source: Microbiome. 2019 Apr 2;7:54. doi: 10.1186/s40168-019-0660-3 (PMC6446388; doi:10.1186/s40168-019-0660-3)
Supplement: Supplementary file 1 — Table S1. Summary of longitudinal microbiome data sets. For each data set, we show the total number of individuals ni, number of time series samples ns, number of microbial taxa reported nt, original sampling rate, and list of clinical attributes available. (PDF 57 kb) [file 40168_2019_660_MOESM1_ESM.pdf]

Table S1

**Data set**	n_i	n_s	n_t	**Sampling**	**Clinical attributes**
Infant gut	58	922	29	Every day or two	Day of life<br>Gestational age at birth<br>Post-conceptional age<br>Gender (female or male)<br>Mode of birth (C-section or vaginal delivery)<br>Room type (single or multi-patient)<br>Human milk used (% of enteral volume provided by human milk)<br>Days of antibiotics (% of days of life on antibiotic)
Vaginal	32	937	330	Twice a week	Day period (days since menses started)<br>Nugent category (low, intermediate , high)<br>Age group ($\leq 30, > 30$ and ≤ 40, or > 40)<br>Race (white, black, hispanic, or other)<br>Tampon used (yes or no)<br>Vaginal douching (yes or no)<br>Sexual activity (yes or no)
Oral cavity	18	374	$1,420$	Every week during gestation	Gestational day of delivery<br>Ethnicity (hispanic or non-hispanic)
