# Supplementary figures and images for: Dynamic interaction network inference from longitudinal microbiome data
Source: Microbiome. 2019 Apr 2;7:54. doi: 10.1186/s40168-019-0660-3 (PMC6446388; doi:10.1186/s40168-019-0660-3)

**a**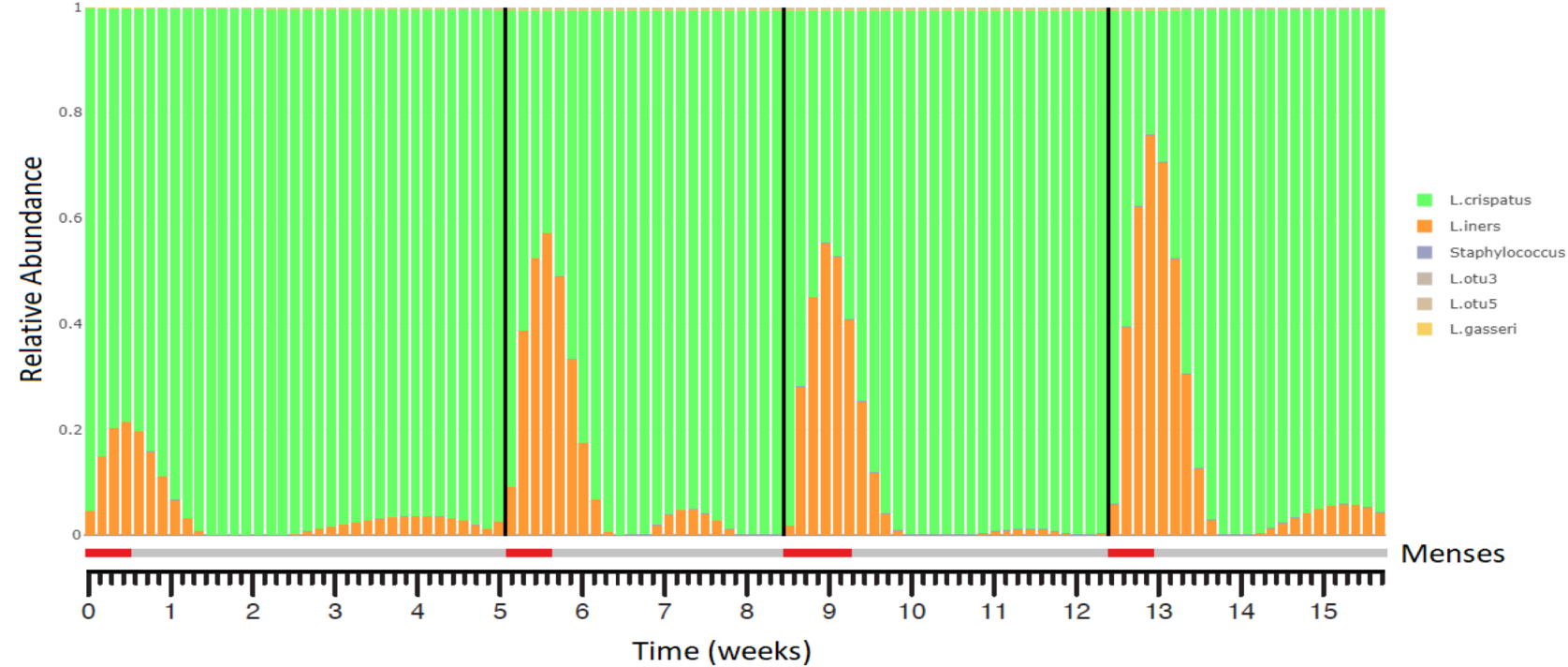**b**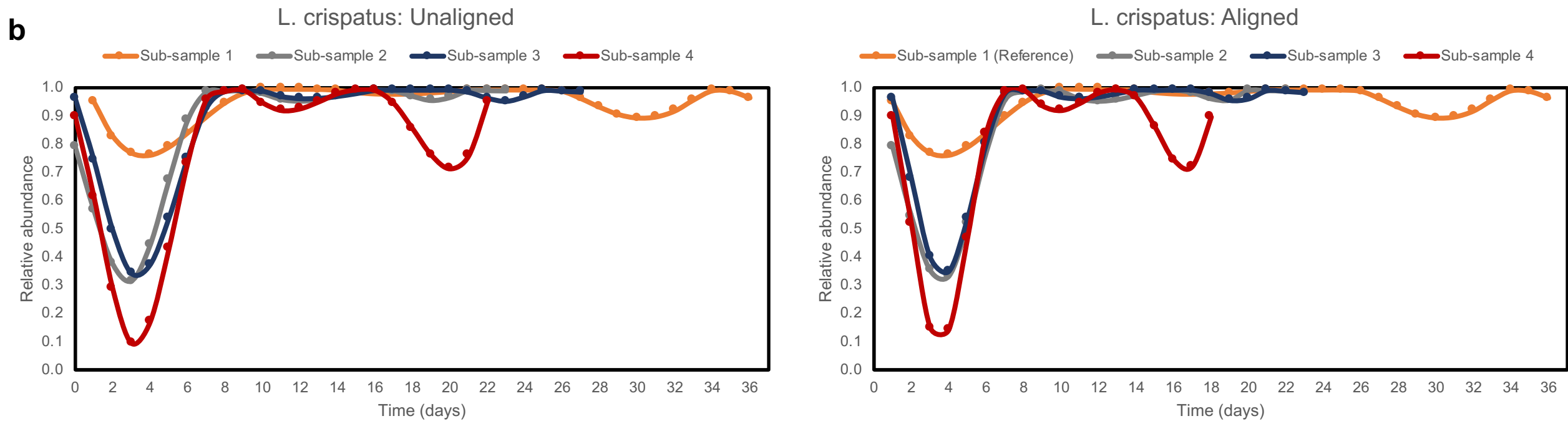

Supplement: Supplementary file 2 — Figure S1. Representative vaginal microbiome sample for subject 28 over the 16-week period. a Relative abundance profile of six vaginal taxa for subject 48 over 16 weeks annotated with menses information. The vertical black lines correspond to the division of sub-samples based on menstrual periods (i.e., 4 sub-samples). Note the interpolated shift in dominance during menses between L. crispatus and L. iners. b | Temporal alignment between the sub-samples from subject 28 time-series data for taxa L. crispatus using the first menstrual period sub-sample as reference (shown in orange). Figure also shows abundance profile of L. crispatus for each sub-sample before (left) and after (right) alignment. (PDF 431 kb) [file 40168_2019_660_MOESM2_ESM.pdf]

**a****Bacilli**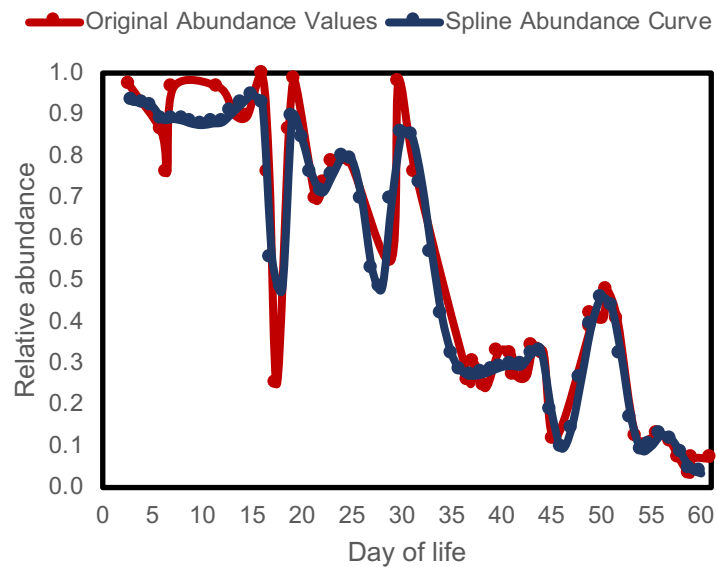

Infant gut

**b****L. iners**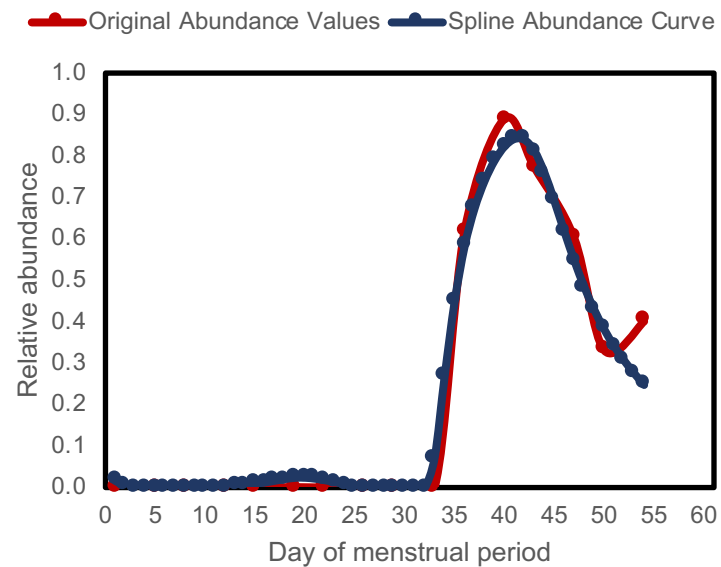

Vaginal

**c****Prevotella**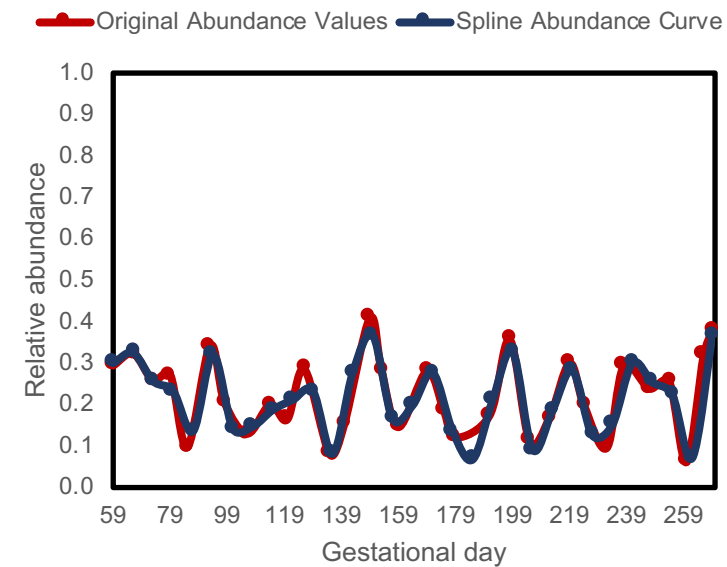

Oral cavity

Supplement: Supplementary file 3 — Figure S2. Original and cubic spline of the abundance profile of a representative microbial taxa for each data set. Figure shows the original abundance values vs. the cubic B-spline curve for a representative taxa profile from a randomly selected individual sample across each data set. aBacilli from the infant gut microbiome. bL. iners from the vaginal microbiome. cPrevotella from the oral cavity microbiome. (PDF 39 kb) [file 40168_2019_660_MOESM3_ESM.pdf]

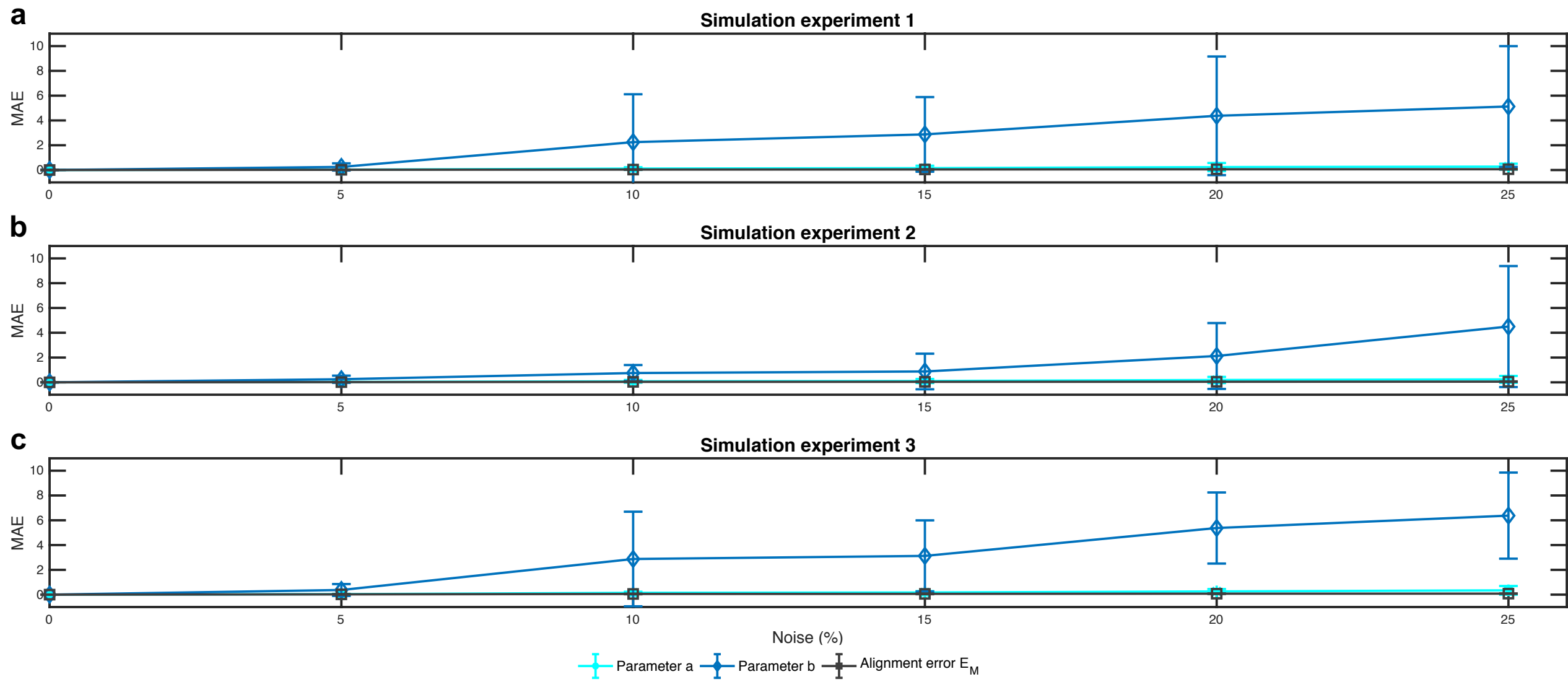

Supplement: Supplementary file 5 — Figure S3. Temporal alignment accuracy on simulated data. Figure shows MAE alongside standard deviation for alignment parameters a and b, as well as alignment error EM using our heuristic alignment approach as a function of percentage of Gaussian noise. a Alignment performance on simulation experiment 1. b Alignment performance on simulation experiment 2. c Alignment performance on simulation experiment 3. (PDF 33 kb) [file 40168_2019_660_MOESM5_ESM.pdf]

**a**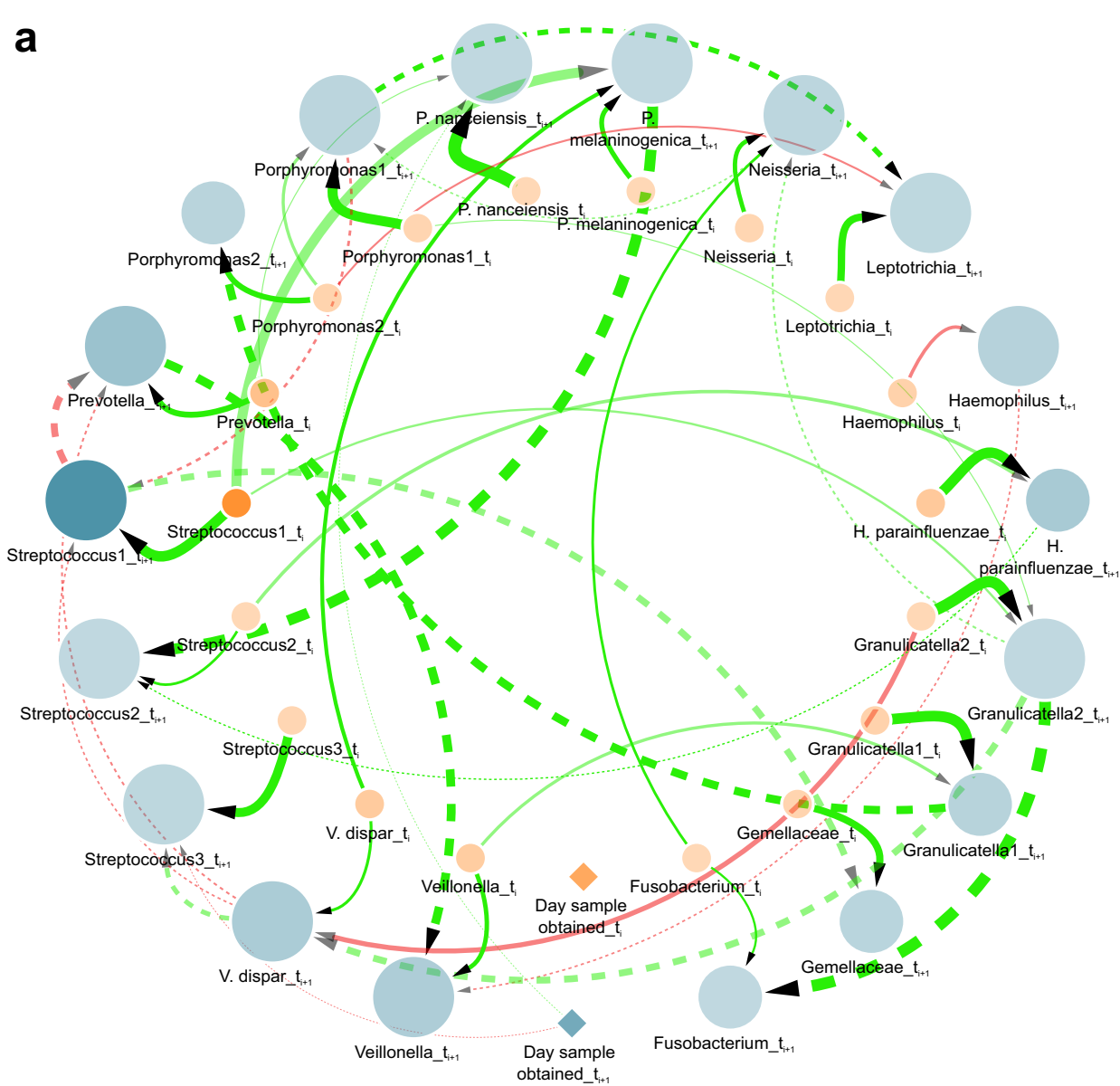

Oral cavity from aligned samples

**b**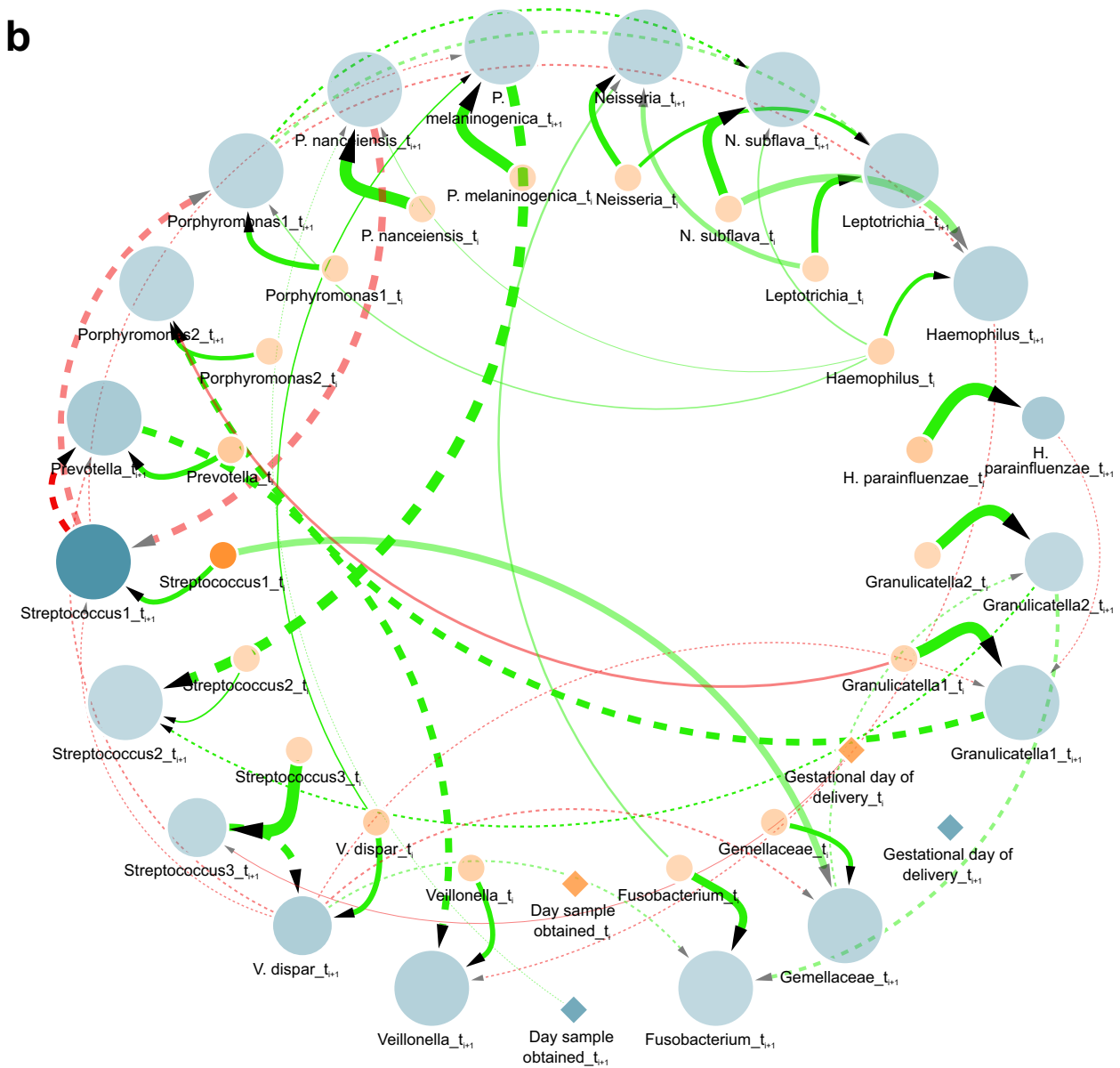

Oral cavity from non-aligned samples

Supplement: Supplementary file 6 — Figure S4. Learned dynamic Bayesian network of the oral microbiome derived from unaligned and aligned tooth/gum samples. Figure shows two consecutive time slices ti (orange) and ti+1 (blue), where nodes are either microbial taxa (circles) or clinical factors (diamonds). Nodes size is proportional to in-degree whereas taxa nodes transparency indicates mean abundance. Additionally, dotted lines denote intra edges whereas solid lines denote inter edges. Edges color indicates positive (green) or negative (red) temporal influence, and edge transparency indicates strength of bootstrap value. Edge thickness indicates statistical influence of regression coefficient as described in network visualization. a Learned DBN for the aligned oral microbiome data at a sampling rate of 7 days and maxParents = 3. b Learned DBN for the unaligned oral microbiome data at a sampling rate of 7 days and maxParents = 3. (PDF 55 kb) [file 40168_2019_660_MOESM6_ESM.pdf]

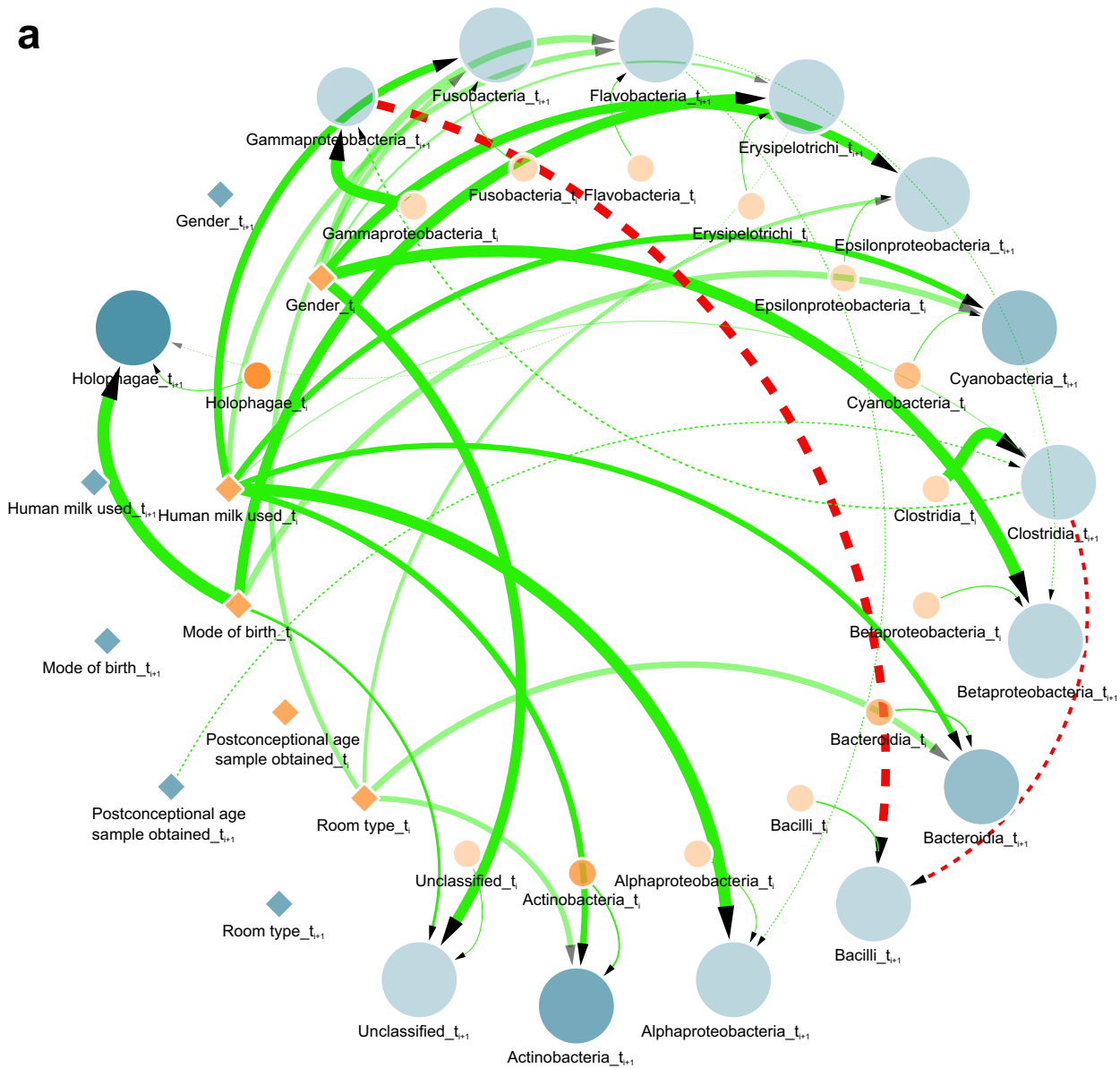

Infant gut from non-aligned samples

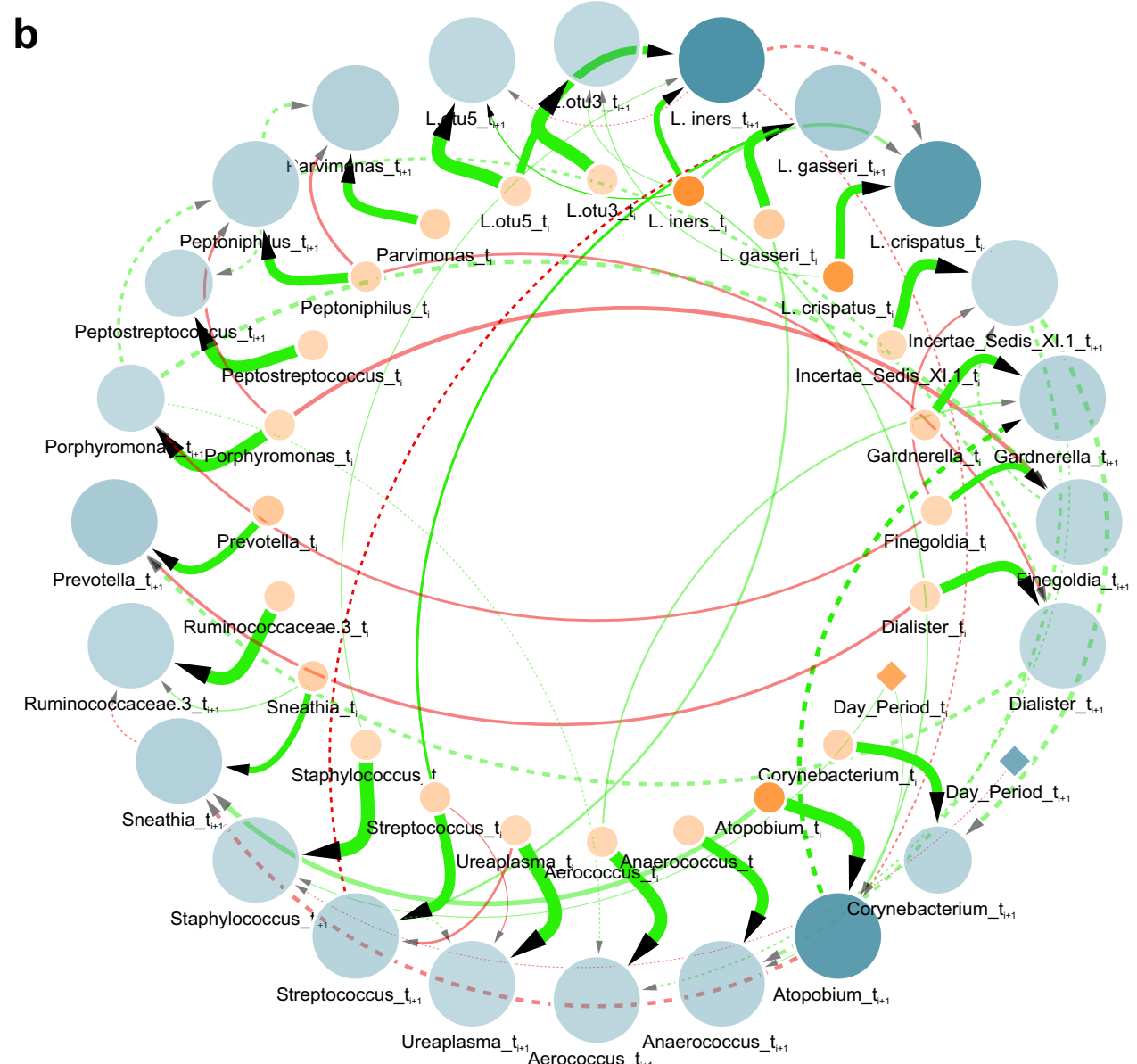

Vaginal from non-aligned samples

Supplement: Supplementary file 7 — Figure S5. Learned dynamic Bayesian network for gut and vaginal microbiomes derived from unaligned samples. Figure shows two consecutive time slices ti (orange) and ti+1 (blue), where nodes are either microbial taxa (circles) or clinical/demographic factors (diamonds). Nodes size is proportional to in-degree whereas taxa nodes transparency indicates mean abundance. Additionally, dotted lines denote intra edges (i.e., directed links between nodes in same time slice) whereas solid lines denote inter edges (i.e., directed links between nodes in different time slices). Edge color indicates positive (green) or negative (red) temporal influence, and edge transparency indicates strength of bootstrap support. Edge thickness indicates statistical influence of regression coefficient as described in network visualization. a Learned DBN for the unaligned infant gut microbiome data at a sampling rate of 3 days and maxParents = 3. b Learned DBN for the unaligned vaginal microbiome data at a sampling rate of 3 days and maxParents = 3. (PDF 56 kb) [file 40168_2019_660_MOESM7_ESM.pdf]

**a**

Infant gut

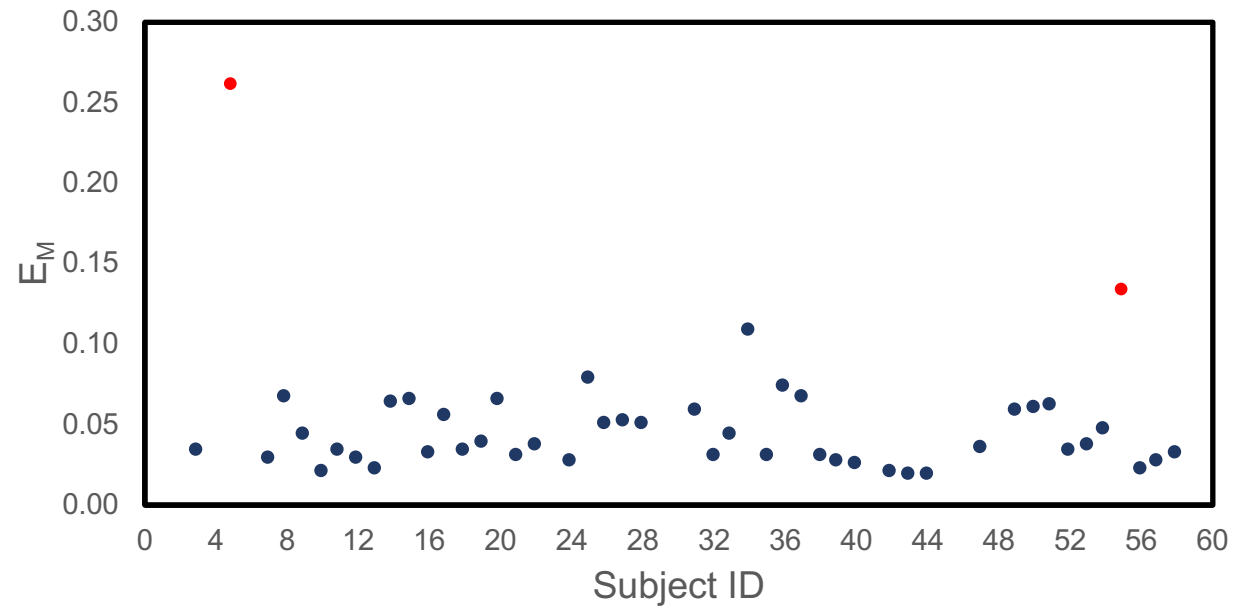**b**

Vaginal

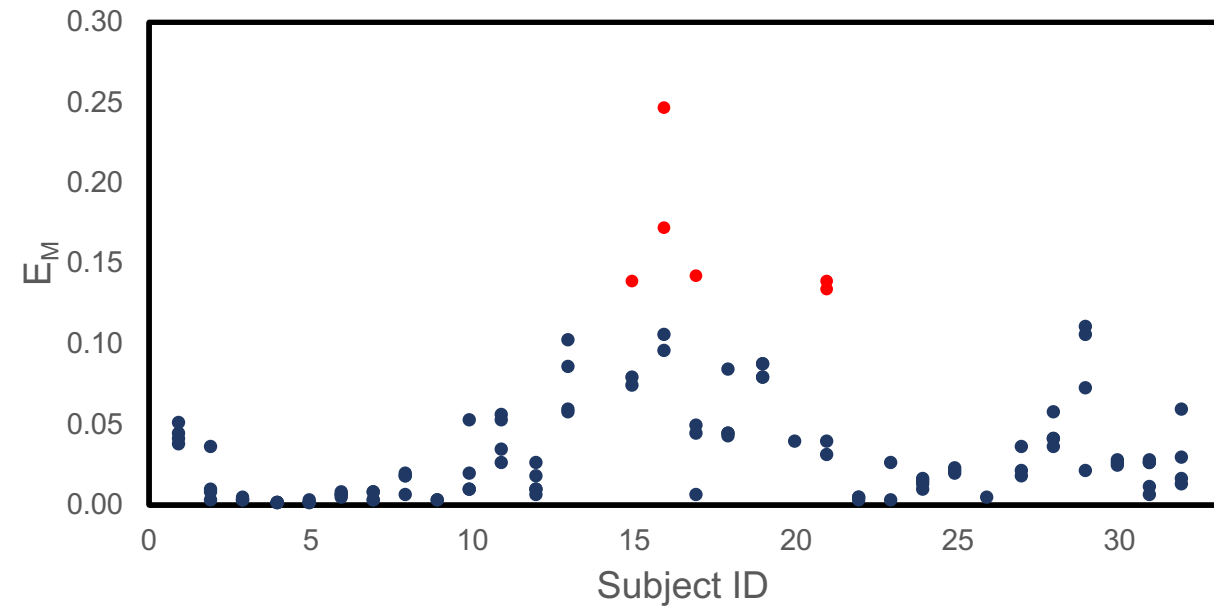

Supplement: Supplementary file 10 — Figure S7. Distribution of microbiome alignment error EM for infant gut and vaginal data sets. a EM scores for 47 infant gut samples aligned against a common reference gut sample. b EM scores for 112 vaginal microbiome sub-samples aligned against an optimal reference sub-sample. In both panels, the scores highlighted in red represent samples with EM at least two standard deviations away from the mean of the distribution of microbiome alignment errors, thus, identified as outliers and removed. (PDF 21 kb) [file 40168_2019_660_MOESM10_ESM.pdf]
