# Supplementary material for: Dynamic interaction network inference from longitudinal microbiome data
Source: Microbiome. 2019 Apr 2;7:54. doi: 10.1186/s40168-019-0660-3 (PMC6446388; doi:10.1186/s40168-019-0660-3)
Supplement: Supplementary file 4 — Table S2. Summary of alignment information. For each data set, we show reference sample, number of aligned samples nr, and selected taxa. (PDF 61 kb) [file 40168_2019_660_MOESM4_ESM.pdf]

Table S2

Data set	Reference sample	n_r	Selected taxa
Infant gut	Subject 48	47	*Actinobacteria*<br>*Alphaproteobacteria*<br>*Bacilli*<br>*Bacteroidia*<br>*Betaproteobacteria*<br>*Clostridia*<br>*Cyanobacteria*<br>*Epsilonproteobacteria*<br>*Erysipelotrichi*<br>*Flavobacteria*<br>*Fusobacteria*<br>*Gammaproteobacteria*<br>*Holophagae*<br>*Unclassified*
Vaginal	Subject 26, Menses 1	112	*Aerococcus*<br>*Anaerococcus*<br>*Ureaplasma*<br>*Parvimonas*<br>*L. iners*<br>*Finegoldia*<br>*Staphylococcus*<br>*Porphyromonas*<br>*Atopobium*<br>*Gardnerella*<br>*L. crispatus*<br>*Peptostreptococcus*<br>*Sneathia*<br>*Streptococcus*<br>*Prevotella*<br>*Peptoniphilus*<br>*Incertae_Sedis_XI.1*<br>*L. gasseri*<br>*Corynebacterium*<br>*Dialister*<br>*L. otu5*<br>*L. otu3*<br>*Ruminococcaceae.3*
Oral cavity	Subject T18	14	*H. parainfluenzae*<br>*Streptococcus*<br>*Gemellaceae*<br>*Porphyromonas*<br>*Streptococcus*<br>*Leptotrichia*<br>*P. nanceiensis*<br>*V. dispar*<br>*Granulicatella*<br>*Veillonella*<br>*Streptococcus*<br>*Neisseria*<br>*N. subflava*<br>*Fusobacterium*<br>*P. melaninogenica*<br>*Haemophilus*<br>*Prevotella*<br>*Porphyromonas*<br>*Granulicatella*
