# Supplementary material for: Dynamic interaction network inference from longitudinal microbiome data
Source: Microbiome. 2019 Apr 2;7:54. doi: 10.1186/s40168-019-0660-3 (PMC6446388; doi:10.1186/s40168-019-0660-3)
Supplement: Supplementary file 8 — Table S3. Summary of average predictive accuracy and standard deviation between methods on the filtered data sets. For each data set, we list the average MAE and standard deviation (presented as percentage) of our proposed DBN models against a baseline method and previously published approaches across different sampling rates. Additionally, each method is run on the non-aligned and aligned data sets. The highest predictive accuracy for each sampling rate is shown in boldface. (PDF 60 kb) [file 40168_2019_660_MOESM8_ESM.pdf]

Table S3

Data set	*sr*	Baseline		McGeachie *et al.*		McGeachie *et al.*++		MTPLasso		Our	
	(days)	non-aligned	aligned	non-aligned	aligned	non-aligned	aligned	non-aligned	aligned	non-aligned	aligned
Infant gut	1	1.87 ± 1.11	1.37 ± 0.80	2.48 ± 1.03	1.97 ± 1.08	1.45 ± 0.76	1.16 ± 1.00	2.34 ± 1.10	1.74 ± 0.83	1.25 ± 0.81	$\mathbf{0.91 \pm 0.70}$
	3	3.84 ± 2.09	2.88 ± 1.21	4.31 ± 1.53	3.38 ± 1.19	1.90 ± 0.94	1.51 ± 0.95	4.06 ± 1.43	3.16 ± 0.96	1.50 ± 0.97	$\mathbf{1.10 \pm 0.80}$
	5	4.79 ± 2.41	4.11 ± 1.52	5.15 ± 1.56	4.25 ± 1.19	2.20 ± 1.05	1.71 ± 1.09	4.86 ± 1.64	4.17 ± 1.13	1.48 ± 1.01	$\mathbf{1.16 \pm 0.93}$
	7	5.07 ± 2.05	4.62 ± 2.21	5.00 ± 1.69	4.65 ± 1.46	1.89 ± 0.90	1.91 ± 1.31	4.92 ± 1.54	4.48 ± 1.37	4.80 ± 1.63	$\mathbf{0.99 \pm 0.96}$
Vaginal	1	0.45 ± 0.17	$\mathbf{0.21 \pm 0.10}$	0.82 ± 0.21	0.61 ± 0.33	0.81 ± 0.23	0.62 ± 0.34	0.76 ± 0.15	0.58 ± 0.18	0.44 ± 0.15	0.24 ± 0.10
	3	1.38 ± 0.42	$\mathbf{0.64 \pm 0.26}$	1.88 ± 0.40	1.22 ± 0.33	1.65 ± 0.50	1.21 ± 0.38	1.66 ± 0.52	1.16 ± 0.27	1.10 ± 0.38	0.65 ± 0.28
	5	2.08 ± 0.58	1.05 ± 0.38	2.64 ± 0.54	1.67 ± 0.39	2.12 ± 0.69	1.63 ± 0.50	2.29 ± 0.68	1.62 ± 0.38	1.67 ± 0.67	$\mathbf{1.02 \pm 0.37}$
	7	2.40 ± 0.76	1.43 ± 0.50	3.00 ± 0.70	2.08 ± 0.48	2.47 ± 0.95	1.97 ± 0.68	2.55 ± 0.80	1.98 ± 0.56	1.90 ± 0.90	$\mathbf{1.28 \pm 0.52}$
	14	3.10 ± 0.77	2.60 ± 0.85	3.78 ± 0.84	3.05 ± 0.78	3.04 ± 1.17	2.66 ± 0.95	3.38 ± 1.11	2.81 ± 0.94	2.40 ± 1.09	$\mathbf{1.91 \pm 1.05}$
Oral cavity	1	0.47 ± 0.11	$\mathbf{0.45 \pm 0.21}$	0.96 ± 0.34	1.11 ± 0.28	0.95 ± 0.40	1.10 ± 0.29	0.96 ± 0.07	1.11 ± 0.19	0.56 ± 0.25	0.57 ± 0.38
	3	1.38 ± 0.29	$\mathbf{1.31 \pm 0.48}$	1.98 ± 0.43	2.11 ± 0.39	1.83 ± 0.49	1.97 ± 0.40	1.92 ± 0.28	2.01 ± 0.22	1.46 ± 0.54	1.34 ± 0.61
	5	2.16 ± 0.40	1.95 ± 0.54	2.66 ± 0.45	2.67 ± 0.44	2.30 ± 0.51	2.40 ± 0.42	2.59 ± 0.58	2.56 ± 0.27	2.09 ± 0.66	$\mathbf{1.92 \pm 0.58}$
	7	2.70 ± 0.51	2.41 ± 0.61	3.11 ± 0.51	3.09 ± 0.52	2.54 ± 0.55	2.66 ± 0.47	2.99 ± 0.64	2.84 ± 0.23	2.44 ± 0.69	$\mathbf{2.18 \pm 0.68}$
	14	3.05 ± 0.68	2.81 ± 0.46	3.44 ± 0.57	3.45 ± 0.64	2.88 ± 0.60	3.10 ± 0.61	3.21 ± 0.49	3.20 ± 0.26	$\mathbf{2.61 \pm 0.74}$	2.89 ± 0.78
	21	3.02 ± 0.47	2.89 ± 0.62	3.43 ± 0.53	3.71 ± 0.62	3.06 ± 0.52	3.27 ± 0.69	3.27 ± 0.59	3.57 ± 0.38	2.67 ± 0.68	$\mathbf{2.50 \pm 0.78}$
	28	3.09 ± 0.81	$\mathbf{2.91 \pm 0.55}$	3.67 ± 0.76	3.88 ± 0.88	3.35 ± 0.82	3.89 ± 0.87	3.44 ± 0.70	3.85 ± 0.29	3.00 ± 0.86	3.28 ± 1.04
