# Supplementary material for: Dynamic interaction network inference from longitudinal microbiome data
Source: Microbiome. 2019 Apr 2;7:54. doi: 10.1186/s40168-019-0660-3 (PMC6446388; doi:10.1186/s40168-019-0660-3)
Supplement: Supplementary file 9 — Figure S6. Comparison of average predictive accuracy and standard deviation between methods on the filtered data sets. Figure shows the average MAE and standard deviation of our proposed DBN models against a baseline method and previously published approaches as a function of sampling rates. Additionally, each method is run on the unaligned and aligned data sets. a Performance results for infant gut microbiome data. b Performance results for vaginal microbiome data. c Performance results for oral cavity microbiome data. (PDF 51 kb) [file 40168_2019_660_MOESM9_ESM.pdf]

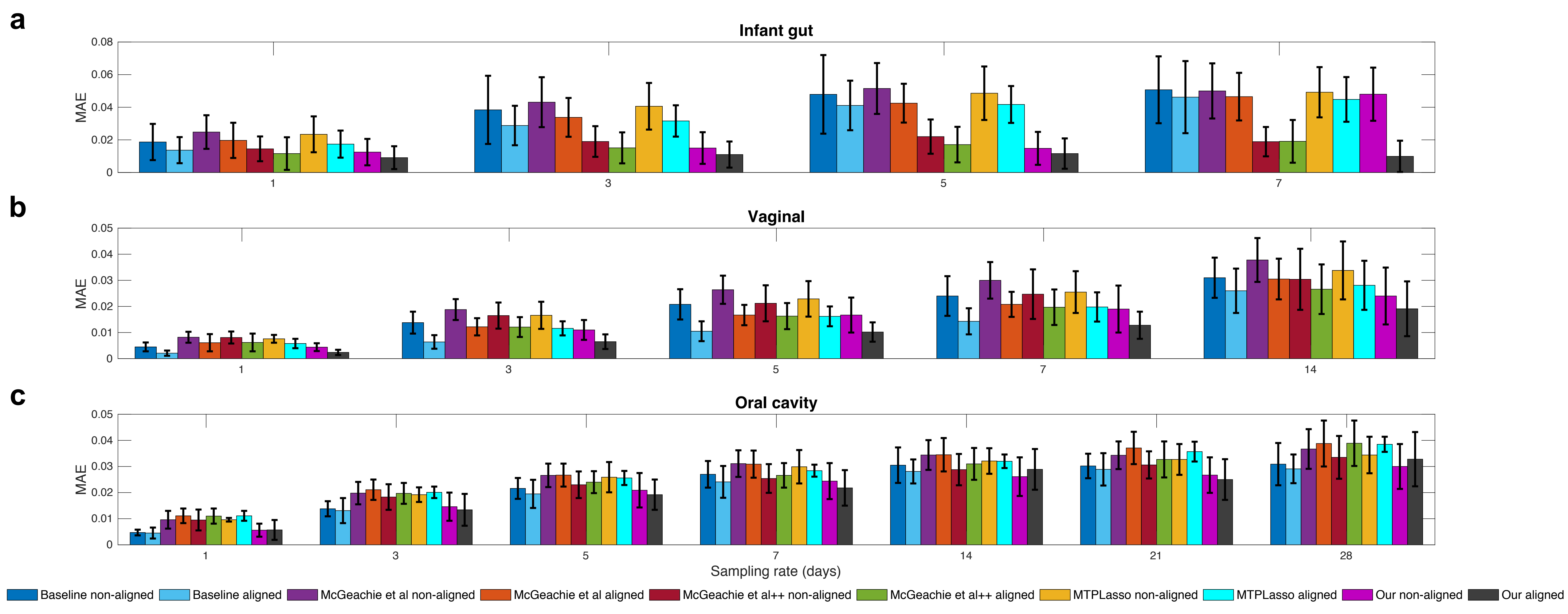
a
Infant gut
MAE
0.08
0.06
0.04
0.02
0
1
3
5
7
b
Vaginal
MAE
0.05
0.04
0.03
0.02
0.01
0
1
3
5
7
14
c
Oral cavity
MAE
0.05
0.04
0.03
0.02
0.01
0
1
3
5
7
14
21
28
Sampling rate (days)
Baseline non-aligned
Baseline aligned
McGeachie et al non-aligned
McGeachie et al aligned
McGeachie et al++ non-aligned
McGeachie et al++ aligned
MTPLasso non-aligned
MTPLasso aligned
Our non-aligned
Our aligned
