# Supplementary material for: Dynamic interaction network inference from longitudinal microbiome data
Source: Microbiome. 2019 Apr 2;7:54. doi: 10.1186/s40168-019-0660-3 (PMC6446388; doi:10.1186/s40168-019-0660-3)
Supplement: Supplementary file 11 — Figure S8. Effect of outliers on average predictive accuracy from aligned data sets. Figure shows the average MAE for our proposed DBN model and baseline method as a function of sampling rates before (labeled as unfiltered) and after (labeled as filtered) removal of outliers. a Performance results for infant gut microbiome data. b Performance results for vaginal microbiome data. (PDF 30 kb) [file 40168_2019_660_MOESM11_ESM.pdf]

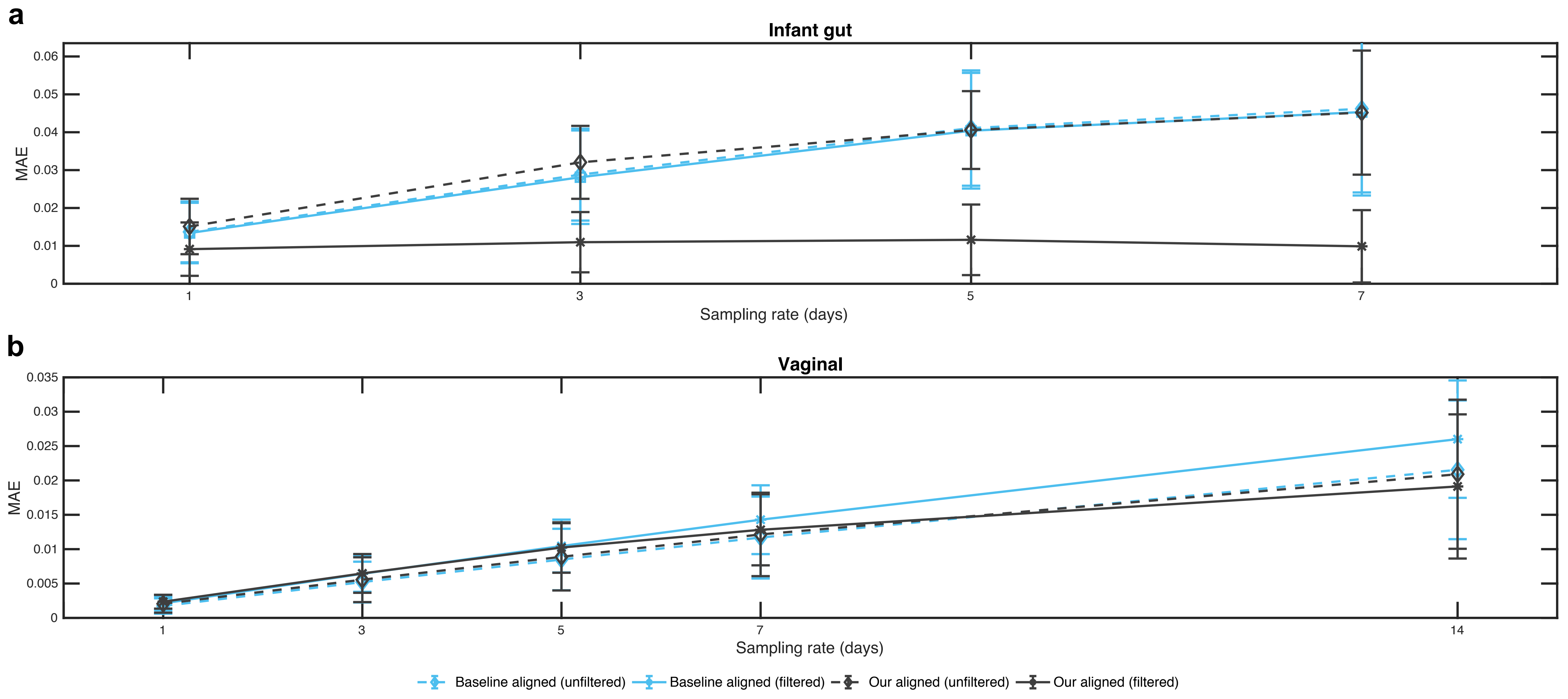
a
Infant gut
MAE
0.06
0.05
0.04
0.03
0.02
0.01
0
1
3
5
7
Sampling rate (days)
b
Vaginal
MAE
0.035
0.03
0.025
0.02
0.015
0.01
0.005
0
1
3
5
7
14
Sampling rate (days)
Baseline aligned (unfiltered)
Baseline aligned (filtered)
Our aligned (unfiltered)
Our aligned (filtered)
